# What is known about muscle weakness, balance impairments and indoor mobility limitations in oculopharyngeal muscular dystrophy? A scoping review

**DOI:** 10.1177/22143602251397052

**Published:** 2025-12-30

**Authors:** Nicolas Bélair, Cynthia Gagnon, Elise Duchesne

**Affiliations:** 1 Faculté de médecine et des sciences de la santé, 7321 Université de Sherbrooke, Sherbrooke, Québec, Canada; 2 Groupe de recherche interdisciplinaire sur les maladies neuromusculaires (GRIMN), Centre intégré universitaire de santé et de services sociaux du Saguenay-Lac-St-Jean, Jonquière, Québec, Canada; 3 Centre de recherche et d’innovation du Centre intégré universitaire du Saguenay-Lac-St-Jean, Clinique des maladies neuromusculaires, Jonquière, Québec, Canada; 4 Centre de recherche du Centre hospitalier universitaire de Sherbrooke (CRCHUS), Université de Sherbrooke, Québec, Canada; 5 CHU de Québec - Centre de Recherche de 4440 l’Université Laval, Québec, Québec, Canada; 6 École des sciences de la réadaptation, Faculté de Médecine, Université Laval, Québec, Canada; 7 Centre Interdisciplinaire de Recherche en Réadaptation Et Intégration Sociale (CIRRIS), Institut de Réadaptation en Déficience Physique de Québec, Québec, Canada

**Keywords:** oculopharyngeal muscular dystrophy, muscle strength, impairments, mobility, limitations, balance

## Abstract

**Objective:**

The objective of this scoping review was to map the body of existing literature regarding muscle strength, balance and mobility limitations in oculopharyngeal muscular dystrophy (OPMD).

**Methods:**

The Joanna Briggs Institute's methodology was followed. All original research including participants with OPMD 18 years or older and describing muscle strength, balance and/or mobility limitations using clinical outcome assessments retrieved from the PubMed, MEDLINE, PEDro, Cochrane Database of Systematic Reviews, and CINHAL Plus databases (searched before January 30^th^, 2023) were included.

**Results:**

Titles and abstracts from 416 studies were screened, and 54 articles were fully reviewed. Proximal upper and lower limb muscle strength were assessed in majority (92.6% of studies). Lack of common methodology made conclusions on their prevalence and severity difficult. Regarding balance, no performance outcome measures was retrieved. 29.6 to 63.0% of studies addressed mobility limitations; only two prospective longitudinal studies were retrieved, and two studies used reference values to assess impairments/limitations severity.

**Conclusion:**

This scoping review highlights the current knowledge on muscle strength, balance and mobility limitations in OPMD. A lack of balance and complete mobility assessments, and OPMD-specific questionnaire for motor impairments or mobility limitations are noted. Future studies should focus on using standardized clinical outcome assessments to better document these impairments/limitations.

## Introduction

Oculopharyngeal muscular dystrophy (OPMD) is a late-onset neuromuscular disease caused by an expansion of (GCN) trinucleotide repetition ranging from 11 to 18 in the *PABPN1* gene, coding for polyA-binding protein nuclear 1 (PABPN1) protein.^1,2^ OPMD is found in several countries, and one of the highest prevalences is found among French Canadians (1/1000)^3^ and Bukhara Jews (1/600).^4^ It is a dominant disease, but rare cases of autosomal recessive inheritance, where individuals inherit two copies of the mutated gene (either as compound heterozygous or homozygous) have been described.^5,6^ The first symptoms appear after 40 years old, and the main symptoms classically reported are ptosis, dysphagia, and muscle weakness, with a proximal-to-distal progression.^7–9^

Although previous research has primarily focused on the cardinal symptom of dysphagia, our research team and others have already documented some major muscle impairments and mobility limitations in OPMD, emphasizing the need to further study impairments related to muscle weakness. A recent study highlighted that mobility is the most affected domain of social participation, and the one where participants expressed the lowest satisfaction level.^10^ Mobility is a very large concept encompassing indoor mobility, defined as the ability to move independently within the home environment by Shumway-Cook, which in turn influences autonomous living.^11^ In OPMD, a cross-sectional study using semi-structured interviews identified issues from a patient's perspective regarding indoor mobility, including difficulty climbing stairs, walking, and getting up from the floor.^12^ Furthermore, our team documented performances in clinical outcome assessments (COAs), including walking speed, sit-to-stand, and stair climbing, below 90.4% of reference values (50.3%–90.4%) for all participants.^9^ In contrast, others have found no difference between OPMD patients and healthy controls while documenting functional mobility with the Timed Up & Go (TUG) or global motor function with the Motor Function Measurement (MFM).^13^

Several factors may influence indoor mobility in OPMD, including lower limb and axial muscle weakness, as well as balance. The largest retrospective study (n = 333) carried out in this population has revealed a high prevalence of proximal lower limb muscle weakness (86.7%) documented with manual muscle testing (MMT).^14^ These results were further supported and expanded by a small cross-sectional study (n = 34) using quantitative hand-held dynamometry.^9^ This study showed that the muscle strength (in percentage of reference values) of two proximal lower limb muscle groups (hip flexors and knee extensors) was below reference values and that this gap seems to increase with age, with older participants being further from reference values than the youngest. Notably, hip flexion strength was the most severely impaired muscle group, with strength below 65.9% of reference values. However, another study has shown no difference in muscle strength between OPMD participants and controls.^15^ Furthermore, although OPMD is often described as a disease affecting proximal muscles, some authors also reported distal and axial muscle weaknesses, which could also influence mobility.^16–18^ Finally, a decline in stance tasks and transfers has been reported over a 20-month period using the MFM,^19^ which may potentially lead to an increase in fall risks, where upper extremity and eyelid muscle weakness could also play a role.

These previous studies highlight the need to better document symptoms of OPMD beyond dysphagia, such as muscle weakness, balance impairments and mobility limitations, to support the development of interventions and better inform patients and healthcare providers about the course of the disease. However, the literature on this topic, along with the COAs used, seems scarce. To adequately measure this gap, there is a crucial need to draw up a comprehensive portrait of the existing literature in this field. The objective of this scoping review was to map the existing literature focusing on lower, upper and axial muscle weakness, balance impairments, and indoor mobility limitations and to document the COAs used to assess them among the OPMD population.

## Material and methods

A preliminary search in the PubMed, MEDLINE, PEDro, the Cochrane Database of Systematic Reviews, and CINHAL Plus databases was performed, and no current systematic or scoping reviews were identified. This scoping study was conducted in accordance with Joanna Briggs Institute's (JBI) methodology for scoping reviews.^20^

The review question was developed using Population, Concept and Context. “oculopharyngeal muscular dystrophy” was the Population, “muscle weakness”, “balance impairments” and “mobility limitations” were the Concepts, and “clinical outcome assessment” was the Context. Moreover, the “muscle weakness” concept was divided into the following sub-concepts: “lower limb proximal muscle weakness”, “lower limb distal muscle weakness”, “upper limb proximal muscle weakness”, “upper limb distal muscle weakness”, and “axial muscle weakness”. Indoor mobility limitations were related to Shumway-Cook^11^ definition. Three sub-concepts for indoor mobility were identified and coded using the International Classification of Functioning, Disability and Health (ICF) terminology^21^:

- **Walking short distances (d4500):** “Walking for less than a kilometer, such as walking around rooms or hallways, within a building or for short distances outside”.
- **Changing basic body position (d410):** “Getting into and out of a body position and moving from one location to another, such as getting up out of a chair to lie down on a bed, and getting into and out of positions of sitting, standing, kneeling or squatting.”
- **Going up and down stairs (d451): “**Moving upwards and downwards so that at least one foot is always on the ground such as ascending and descending stairs or curbs.”

Therefore, the research question was: “What is known in oculopharyngeal muscular dystrophy about muscle weakness, balance impairments, and indoor mobility limitations”?

### Search strategy

Thesaurus keywords and synonyms of the population and the chosen concepts were identified according to each selected database. The abbreviated version of the terms is as follow: (“Oculopharyngeal muscular dystrophy” OR (“muscular dystrophies” [MeSH Terms] AND “oculopharyngeal” [All fields])) AND (motor impair* OR muscle OR mobilit* OR Balance OR “Functional capacit*”) AND human. A detailed version of the terms is available in *Appendix A.* The search strategy aimed to locate studies published in PubMed, MEDLINE, PEDro, Cochrane for systematic reviews databases and CINHAL Plus. Studies published in English and French were included in this scoping review. In addition, relevant articles from the reference list of the selected articles were also added to the final identification of articles. The literature search took place on 2023-01-31.

### Inclusion criteria

This scoping review considered original research without limitation of publication date, including: 1) participants 18 years or older, with genetically or clinically confirmed OPMD (either heterozygous, compound heterozygous or homozygous mutations with (GCN) trinucleotide repeats ranging from 11 to 18); 2) describing either muscle strength, balance, and/or indoor mobility (as defined above) assessed by clinical outcome assessments used in a clinical context. Magnetic resonance imaging (MRI) was not included as COA. The scoping review included all research designs.

### Study selection

Study selection was independently performed by NB, a master's student with four years of experience in neuromuscular research and practicing physiotherapy for one year, and CG, a certified occupational therapist and full professor with extensive experience in neuromuscular research and COAs. All identified studies were transferred to the reference management software EndNote X9.3.3 (Clarivate Analytics, PA, USA) and the Rayyan—AI-powered tool for systematic literature reviews software^22^ to remove duplicates, if any. The full texts were compared to the selection criteria by the two independent reviewers. In the event of further exclusion following the reading of the full text, the reasons for the exclusion were recorded and reported in the Preferred Reporting Items for Systematic Reviews and Meta-analyses extension for Scoping Reviews (PRISMA-ScR) flowchart.^23^ All disagreements during each stage of the selection process were resolved through discussion or the intervention of a third reviewer (ED). The results are presented in a diagram format using the PRISMA-ScR, available in *Appendix B*.

### Data extraction

Data was extracted using a standardized extraction grid. Data extraction on five articles was performed using a preliminary extraction grid. The grid was reviewed and modified by CG to generate the final grid. An overview of the final extraction grid is provided in *Appendix C.* For the extraction of the COAs related to the concepts and sub-concepts, they were divided using the Food & Drug Administration (FDA) categorization for COAs^24^: Patient-reported outcome measures (*PROM*), clinician-reported outcome measures (*ClinRO*) and performance outcome measures (*PerfO*). Their definition is as follows: 1) a *PROM* is defined as: “A measurement based on a report that comes directly from the patient about the status of the patient's health condition without interpretation of the patient's response by a clinician or anyone else”. Self-reported questionnaires were considered *PROM. *In addition, for the scoping review purpose, structured or semi-structured interviews were also included in this category. 2) A *ClinRO* is defined as: “A measurement based on a report that comes from a trained health care professional after observation of a patient's health condition”. The term “Clinical Observation” was chosen for this scoping review to include medical/physician examination from a routine medical visit or when studies did not define the method used to assess the corresponding concept. In addition, patient file review was included as well as visual gait analysis. 3) A *PerfO* is defined as: “A measurement based on a standardized task(s) performed by a patient that is administered and evaluated by an appropriately trained individual or is independently completed”. The *PerfO* category is presented with the most common tests assessing directly the concepts and sub-concepts. Those less frequently used were classified as “other tests”. The data extraction was organized by sub-concepts and COA categories and reported in Supplementary Table S1, Tables 1–9 in a cross-table, indicating the number of articles per category. The studies were classified by concepts (muscle strength, balance, and indoor mobility) and results are presented in Supplementary Tables S2, Tables 1–9 and include: COA categories, outcome, method and findings. Spider graphs present the distribution of study designs and COA categories of sub-concepts and the number of studies per COA. Except for five studies (which are underlined in Supplementary tables),^25–29^ case studies were not included to report the prevalence of a concept and were reported using pale grey text accordingly in all Supplementary Tables. Also, extreme values (minimum and maximum values found in all articles of a category) are reported using **bold green text**.

## Results

### Data analysis and presentation

**Figure 1.**
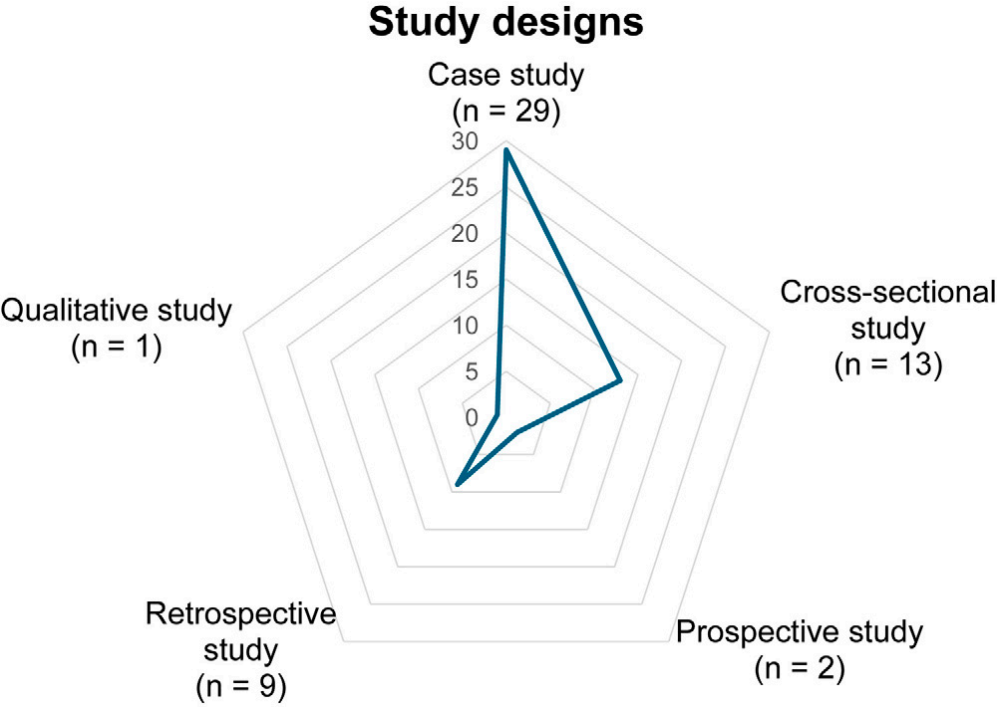
Study designs distribution.

The literature search identified 416 articles, of which 54 remained eligible based on inclusion criteria (see *Appendix B*): 29 were case studies (53.7%), 13 used a cross-sectional design (24.1%) and two were prospective longitudinal studies (3.7%) (Figure 1). All information related to the raw data extracted from the studies can be found in Supplementary Table S3. A summary of findings is presented in Table 1. The studies sample size ranged from 1 to 333 participants with 29 studies (53.7%) having less than 20 participants. The most common COAs were *ClinRO* (clinical observation), followed by *PerfO*. A summary of the sub-concepts and their related COA is presented in Supplementary Table S1, Tables 1–9.

**Table 1.** Documentation of muscle weakness, balance impairments and mobility limitations with related clinical outcome assessments in OPMD.

	Patient-reported (PROM)		Clinician-reported (ClinRO)			Performance (PerfO)		
	Interview	Questionnaire	Clinical observation	Patient file review	Other	Common tests		Other tests
*Presence/progression of muscle weakness*						MMT	QMT	Others
LL Proximal	Yes^12^	Yes^15,^^30–32^	Yes^16,17,25–29,33–46^ No^47–49^	Yes^4,14,50–54^		Yes^15,30,39,41,43,45,^^53–62^ No^48^ Decline^19,63^	Yes^9,13^ No^15^ Decline^19,63^	
LL Distal		No^15,30^	Yes^16–18,25,29,33,40,41,45,46,56,61,62,64^ No^34,39,43,48,49,65^	Yes^53,54^ No^52^ N/R^14^		Yes^15,30,41,45,53,54,60,61^ No^39,43,48,56,58,59^ Decline^19,63^ N/R^14^	Yes^13^	
UL Proximal	Yes^12^	Yes^15,^^30–32^	Yes^13,16,17,25–29,33,34,36–49,56,57,62,64,66,67–69^ No^44,48,49^	Yes^4,14,50–54^		Yes^30,39,41,43,45^^53–62^ Decline^19,63^	Yes^9,13^ Decline^19,63^	Yes^32^
UL Distal	Yes^12^	No^15,30^	Yes^16,17,25,29,31,33,40,41,45,55^ No^34,39,43,48,49,58,59,61,65,67^	Yes^53,54^ No^52^ N/R^14^		Yes^15,30,41,53–55,61^ No^39,43,45,56,58,59,61^ Decline^19,63^	Yes^9,13^ No^32^	
*Axial weakness*	Yes^12^	Yes^15,30,31,60^	Yes^13,15–17,25,27–49,56–62,64,66,68–71^	Yes^4,14,50–54^		Yes^13,30,43,52–60^ Decline^19,63^	No^13^	Yes^32^
*Presence/progression of balance impairments*						Common balance tests		Others
Balance	Yes^12^		Yes^17,45^					Yes^19,35,63^ No^15^ Decline^19,63^
*Presence/progression of mobility limitations*						Common walking/transfer/stair tests		Others
Walking	Yes^12^	Yes^15,30–32,36^	Yes^15–17,28,32,34,37–39,45,49,51,54,56,62,66^ No^29,43,44,57^	Yes^14,51,53,54,69^	Yes^12–14,32,33,36,53,54,62,65,69^	Yes^9^ No^15^		No^15,35^ No decline^19,63^
Changing basic body positions	Yes^12^	Yes^30,32^	Yes^16,17,30,32,34,37,39,61,65^	Yes^14^		Yes^9^		Yes^19,35,63^ No^15^ Decline^19,63^
Going up and down stairs	Yes^12^	Yes^30,32^	Yes^16,28,37–39,43,56,62,66^	Yes^14^		Yes^9,19,63^ Decline^19,63^		

*Note:* “Yes” and “No” refer to the presence or absence of muscle weakness or balance/mobility limitations, as reported in the studies cited. “Decline” refers to a significant progression over time in longitudinal studies. Abbreviations: LL: lower limb; MMT: manual muscle testing; N/R: not reported; QMT: quantitative muscle testing; UL: Upper limb.

*Sociodemographic data.* All the information mentioned in this section is available in Supplementary Table S3. Most studies were from the USA, Netherlands and Japan. A total of 22 countries were represented and each had between one to nine studies. The data presented below exclude case studies, except for five studies^25–29^ which included many cases (more than 29 cases). Among the studies, the lowest average participant age was 42.0 years,^62^ and the highest average age was 66.2 years.^36^ The lowest male proportion was 25.0%^35^ and the highest was 65.3%.^51^ The mean or median reported age at first symptom onset for one of the three cardinal symptoms in OPMD ranged from 43.3^27^ to 63.0 years.^51^ The mean or median of symptom or disease duration ranged between 7.0 years^50^ to 20.0 years.^35^ One study mentioned a median latency of other symptoms before the onset of proximal weakness, which varies between 4.0 years (range 0–21) for the combination of ptosis and dysphagia, 7.0 years (range 0–21) for ptosis and 6.0 years (range 0–25) for dysphagia.^14^

**Figure 2.**
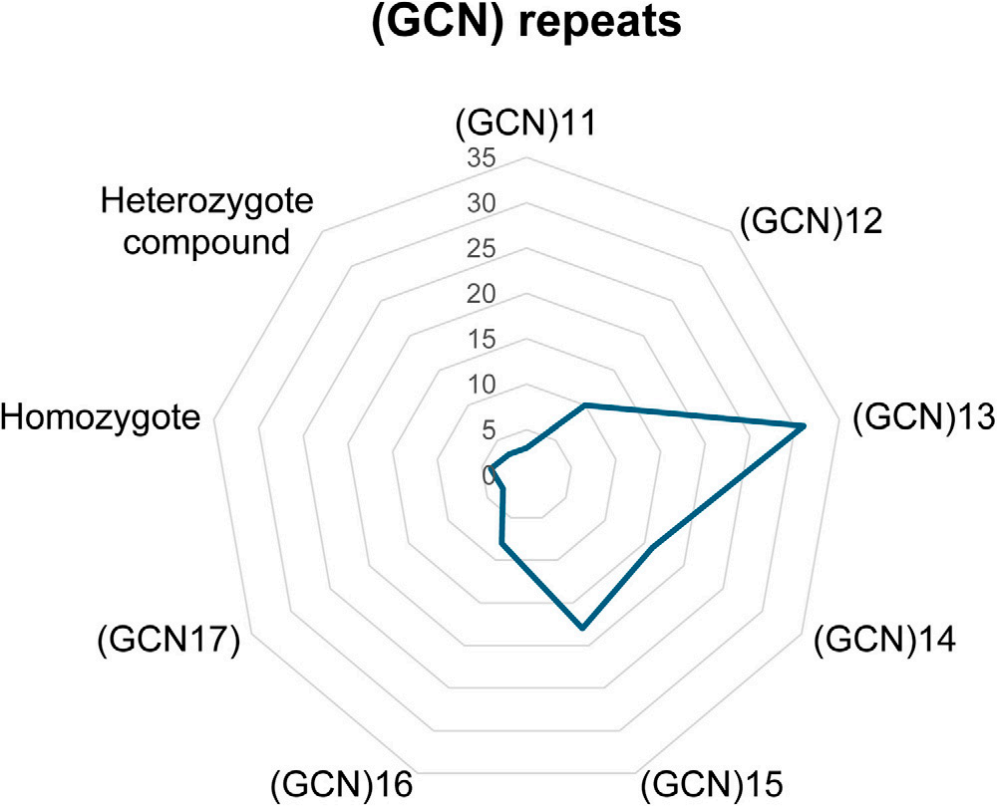
Distribution of studies according to the (GCN) repeat mutation.

*(GCN) repeats.* Heterozygote, heterozygote compound and homozygote mutations present in patients from included studies are shown in Figure 2. Most studies (57.4%) presented heterozygote (GCN) repeats mutation of (GCN)13 (i.e., one “wild type” allele and one mutant allele). Studies using the old nomenclature (only including the PABPN1 gene expansion, i.e., (GCN)6 to (GCN)8-13) were transformed using the new nomenclature (also including the polyalanine stretch in the N-terminal domain of PABPN1, i.e., (GCN)11 to (GCN)18) according to the corresponding number of repetitions in the mutated allele.^70^ Some studies have included participants with different repeat sizes.

### Motor impairments

#### Lower limb muscle weakness

**Figure 3.**
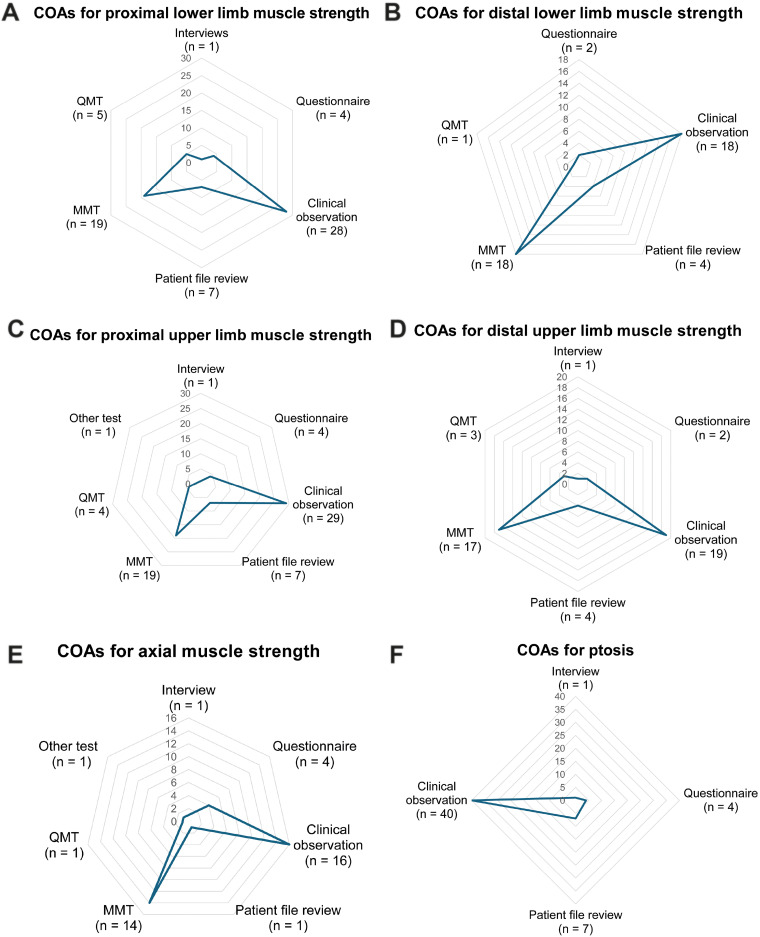
Distribution of COAs across studies for A) proximal lower limb muscle strength, B) distal lower limb muscle strength, C) proximal upper limb muscle strength, D) distal upper limb muscle strength, E) axial muscle strength, and F) ptosis per COA.

Proximal (hip, thigh or knee) lower limb muscle strength was assessed in 50 studies (92.6%), while 31 studies (57.4%) reported on distal (ankle or toes) lower limb muscle strength (Figures 3A and 3B). According to proximal lower limb weakness (Supplementary Tables S1, Table 1 and S2, Table 1), it was reported in 42.9%^15^ to 90.9%^32^ of patients when using a *PROM* in five studies.^12,15,30–32^ Using a *ClinRO* (clinical observation, patient file review), proximal lower limb muscle weakness prevalence ranged from 9.7%^26^ to 86.7%^14^ of patients in 35 studies.^4,14,16,17,25–29,33–51,53–56,62,64,66^ *PerfO* was used in 22 studies to assess proximal lower limb muscle strength.^9,13–15,18,19,30,39,41,43,45,54–60,62,63,67^ Manual muscle testing (MMT) was the most frequent *PerfO* used in 19 studies,^14,15,18,19,30,39,41,43,45,53–59,62,63,67^ as compared to five studies using quantitative muscle testing (QMT).^9,13,15,19,63^ For MMT, when proximal lower limb muscle weakness was present, it was in majority symmetrical and ranged from severe (MRC 2) to mild (MRC 4-4+) in 30.6%^53^ to 85.0%^18^ of patients and was reported using a composite score in three studies.^19,62,63^ For QMT, studies showed discordant results. No weakness for the quadriceps (knee extension) was found in one study,^15^ while two studies showed weaknesses when reported in percentage from reference values according to age and sex for the total sample, reaching 50.0% for hip flexors to 74.0% for knee extensors.^9,13^ For the total sample, no weakness was found for hip extensors and knee flexors in one study,^9^ while another found percentage from reference values ranging approximately from 55.0 to 57.5% for these muscle groups.^13^ Two longitudinal prospective studies showed a range of significant deterioration of muscle strength for hip flexors and knee extensors (−12.2 to −14.3%) over a period of 20 months.^19,63^ While hip flexors and knee extensors were always reported in the studies using QMT, the other mobility-related muscle groups that are reported vary across studies, both for proximal (hip extensors, hip abductors, hip adductors, hip internal and external rotators, knee flexors) and distal lower limb (dorsiflexors, plantar flexors, ankle invertors and evertors).^9,13,15,19,63^ In these studies, standardizations and dynamometers were different.

Regarding distal lower limb strength (Supplementary Table S1 table 2 and S2 table 2), 22 studies using a *ClinRO* (patient file review, clinical observation) reported on it and weakness was present in 3.8^54^ to 32.5%^30^ of patients.^14,16,17,25,29,31,33,34,39–41,43,45,48,49,53–56,62,64,66^ Using a *PROM*, no distal lower limb weakness was reported in two studies.^15,30^ Using a *PerfO*, 19 studies^13–15,18,19,30,39,41,43,45,48,54–57,59,60,62,67^ assessed distal lower limb muscle strength. From these, MMT was used in 18 studies^14,15,18,19,30,39,41,43,45,48,53–56,59,60,62,67^ while QMT was used in one study.^13^ When distal lower limb weakness was present, MMT ranged from moderate (MRC 3) to mild (MRC 4) in 3.8^54^ to 32.5%^18^ of patients and was reported using a composite score in two studies.^19,63^ The only study that used QMT reported percentages from reference values of the ankle muscles of approximately 60.0% for plantar flexors and ankle dorsiflexors.^13^

#### Upper limb muscle weaknesses

For the upper limbs, 50 (92.6%) and 34 (63.0%) studies assessed proximal (shoulder and elbow muscles) (Supplementary Tables S1 table 3 and S2 table 3) and distal (wrist and hand muscles) (Supplementary Tables S1, table 4 and S2, table 4) upper limb muscle strength, respectively (Figures 3C and 3D).

When using a *PROM*, proximal upper limb weakness was reported in 25.7%^31^ to 81.3%^30^ of patients in five studies.^12,15,30–32^ Using a *ClinRO* (clinical observation, patient file review), proximal upper limb weakness was present in 3.6%^14^ to 85.7%^15^ of patients in 36 studies.^4,14,16,17,25–29,33,34,36–51,53–57,62,64,66,68^ When using a *PerfO*, proximal upper limb muscle strength was assessed with MMT in 19 studies,^14,15,18,19,30,39,41,43,45,53–59,62,63,67^ with QMT in four studies^9,13,19,63^ and with a homemade test (classified as “other test”) in one study.^32^ Using MMT, weakness severity ranged between severe (MRC 2) to no weakness (MRC 5) in 5.4%^54^ to 55.0%^18^ of participants. MMT was reported as a composite score in three studies.^19,62,63^ Using QMT to assess proximal upper limb muscle strength, one study showed a significant difference between three age groups for shoulders flexors strength, but not for shoulder abductors and elbow flexors.^9^ In another study, percentages from reference values with matched-age and sex subjects varied approximately between 61.0% to 96.0% of the normative value for shoulders abductors, elbow flexors and elbows extensors and ranged between less than one standard deviation to more than two standard deviations in comparison to a control group.^13^ In two studies, a significant decline over a period of 20 months was found for deltoids muscles (shoulder abductors), ranging from −23.5% (−16.8 to −30.2) for the left side to −26.8% (−16.7 to −36.9) for the right side.^19,63^ Finally, using a *PerfO* categorized as “other test”, an inability to raise the left, right or both arms for 10 s were present in 18.2% of participants.^32^

Using a *PROM*, distal upper limb muscle strength was assessed in three studies,^12,15,30^ from which only one study reported hand weakness in 4.0% of patients.^12^ Distal upper limb weakness using a *ClinRO* ranged from 5.3%^54^ to 25.0%^18^ of patients in 23 studies.^14,16–18,25,29,33,34,39–41,43,45,48,49,53–56,59,60,62,64,66^ Using a *PerfO*, MMT was used in 17 studies^14,15,18,19,30,39,41,43,45,53–56,59,60,62,67^ while QMT was used in three studies.^9,13,32^ Using MMT, weakness ranged between moderate (MRC 3) to no weakness (MRC 5) in 5.3%^54^ to 25.0% of patients.^18^ MMT was reported using a composite score in two studies.^19,63^ Regarding QMT, mean percentage from reference values ranged from 67.1% for grip strength to 73.3% for pinch strength, with a significant difference between three age groups in two studies.^9,13^ In one study, 53.8% of patients had a grip strength reduced by ≥ 1 standard deviation from the control group.^13^ Dynamometer types were different between all studies.

#### Axial weakness

As shown in Supplementary Table S1, Table 5 and S2, Table 5, 32 studies (59.3%) assessed axial (facial, head, trunk and abdominal) muscle strength, while 50 studies (92.6%) assessed ptosis or external eye muscle weakness (Figure 3E and 3F). When using a *PROM*, axial muscle strength was reported in five studies. From these, cervical muscle weakness was reported in 20.0%, facial muscle weakness in 8.0% and eyes muscle weakness (including ptosis) in 76.0% from one study using semi-structured interview,^12^ while the remaining four studies using a *PROM* reported ptosis in up to 49.0%^31^ of patients.^15,18,30,31^ Axial muscle weakness was frequently reported using *ClinRO* (clinical observation, patient file review) when present. When excluding ptosis (marked with a black “x” in the “clinical observation” and “patient file review” columns in Supplementary Table S1, Table 5 and reported using pale grey text in Supplementary Table S2, Table 5), axial muscle weakness ranged from 12.5%^37^ to 81.3%^13^ of patients in 17 studies (10 case studies),^4,13,17,25,27–29,33,37,39,41,43,44,48,49,62,66^ with facial weakness being the most reported. Axial muscle strength (excluding ptosis) was assessed using a *PerfO* with MMT in 14 studies.^13,15,18,19,30,43,53–59,67^ Two studies used a composite MRC score to report their results regarding neck flexors and neck extensors muscle strength.^19,63^ For MMT, strength of neck muscles (neck flexors and extensors) ranged between severe (MRC 2) and no weakness (MRC 5) in 4.0%^56^ to 47.5%^18^ of participants.^13,15,18,19,30,43,53–59,67^ The weakness was more prevalent in neck flexors (33.3%) than extensors (11.1%) in one study.^54^ Facial muscle strength ranged from moderate (MRC 3) to mild (MRC 4) in 12.0%^56^ to 84.6%^13^ of participants in four studies.^13,18,56,57^ Axial muscle strength was assessed using QMT with dynamometry of the neck muscles in one study.^13^ No weakness was reported in neck extensors and flexors when compared to age and sex-matched patients.^13^ One study used a homemade *PerfO*, categorized as “other test”, which showed incapacity to raise the head from bed for 5–6 s in 4.6% of patients.^32^ Standardization and selection of key muscle groups for eyes, head, trunk and abdominal muscles were different among the studies. No assessment of trunk and abdominal muscles was made using QMT.

### Balance impairments

**Figure 4.**
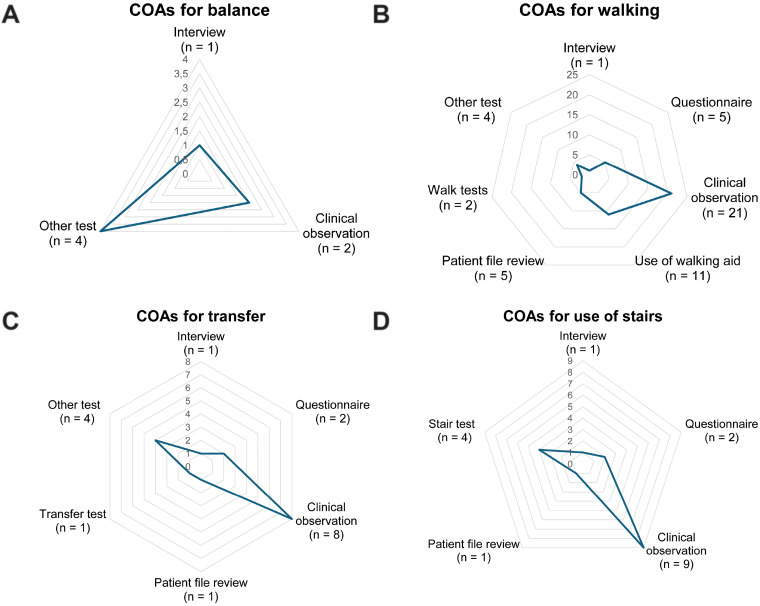
Distribution of COAs across studies for A) balance, B) walking short distances limitations, C) changing basic body position limitations, and D) going up and down the stair limitations.

Seven studies (13.0%) assessed balance impairments (Supplementary Table S1, Table 6 and S2, Table 6) with the COA distribution reported in Figure 4A. When using a *PROM*, “poor balance” was reported in 28.0% of patients in one study using a semi-structured interview.^12^ Regarding *ClinRO*, incapacity to stand, raise to toes/heels or jump on one leg independently was reported through a clinical examination in two case studies.^17,45^ A *PerfO* categorized as “other test” was used in four studies.^15,19,35,63^ The COA selected was identical among these studies (MFM) and results were reported using the total score or subscale score for analysis. The score obtained in part D1 (standing and transfer tasks) was significantly lower than part D2 (axial and proximal limbs motor function) and D3 (distal limbs motor function) in one study,^35^ while no significant difference was found in the total MFM score between patients and control group in another study.^15,35^ Over a period of 20 months, significant decline of part D1 of the MFM was found among participants in two studies.^19,63^ No balance-specific *PerfO* was found in the studies.

### Mobility limitations

#### Walking short distances

34 studies (63.0%) assessed walking short distances limitations (Supplementary Table S1 table 7 and S2 table 7; Figure 4B). When using a *PROM*, difficulty in walking a short distance was reported in 9.2%^32^ to 69.0%^31^ of patients using semi-structured interview or questionnaires in a total of six studies.^12,15,30–32,36^ Using a *ClinRO*, walking limitations ranged between 6.3%^30^ to 42.9%^15^ of participants in 27 studies.^12–14,16,17,28,29,32–34,36–39,43–45,49,51,54–56,58,62,63,66,72^ Among these, one study reported an incapacity to walk a short distance (defined as < 20 meters) in 17.1% of their population using patient file review.^14^ Use of walking aid was reported in 12.0%^69^ to 44.9%^36^ of patients among 11 studies.^12–14,32,33,36,54,55,63,66,72^ Walking limitations were assessed using a *PerfO* in five studies.^9,15,19,35,63^ The 6-min walk test (6MWT) had percentages from reference values between 60.1% to 71.5% in one study (mean = 67.3%), with significant differences between three age groups (≤55y, 56–65y, ≥ 66y).^9^ On the contrary, no significant difference between patients and controls was found in another study.^15^ Walking speed was assessed using the 10-meter walk test (10mWT) in one study^9^ using comfortable and maximal speeds. No significant difference between the three age groups was found for comfortable speed, while a significant difference was found for maximal speed. In the same study, comfortable speed on the 10mWT had percentages from reference values between 83.9% to 95.0% (mean = 90.4%) among the three age groups,^9^ but no comparisons to reference values for maximal speed were available. Four other studies used other *PerfO* tests,^15,19,35,63^ in which no significant difference between patients and controls was found for the actometer^15^ and performances at the MFM total score and subscales.^15,35^ One study showed a significant higher time between OPMD patients and controls to complete the TUG, but mean value was still within normative range.^15^ Part D2 of the MFM (axial and proximal limb motor function) showed a non-significant decline over a period of 20 months in two studies. Walking capacity limitations definitions were different among studies (gait problem,^34^ waddling gait,^16^ use of cane or wheelchair,^33^ gait disturbance,^38^ waddling motion of the pelvis,^37^ walking difficulties^15^ and slow walk due to fatigue^49^).

#### Changing basic body position

Limitations in changing basic body positions (including transfers) (Supplementary Table S1 table 8 and S2 table 8) were reported in 16 studies (29.6%) (Figure 4C). When using a *PROM*, transfer capacity limitations were present in 8.0%^12^ to 54.6%^32^ of patients in three studies.^12,30,32^ Using a *ClinRO*, changing basic body positions was assessed in nine studies^14,16,17,32,34,37,39,61,65^ and ranged from 10.1%^34^ to 59.1%^32^ of patients. Gower's sign test was positive in up to 3/16 participants (18.8%) in three studies.^16,34,61^ Using patient file review, transfer capacity was impaired when assessing the crouch to standing position in 35/320 participants (10.9%)^14^ and Gower's sign test was positive in 97/294 participants (33.0%). Transfer capacity limitations were assessed with a *PerfO* in five studies.^9,15,19,35,63^ One study used a transfer test and showed a significant difference between three age groups for the 30s sit-to-stand test (30s-STS)^9^ and percentages from reference values ranged between 33.4% to 65.7% (mean = 50.3%).^9^ Four studies used the MFM,^15,19,35,63^ with three of them showing a significantly lower score for part D1 (stance and transfer tasks) when compared to parts D2 (axial and proximal limb motor function) and D3 (distal limb motor function).^19,35,63^ A significant decline over a period of 20 months was found only for part D1 of the MFM in two studies.^19,63^ In contrast, one study found no significant difference between patients and control groups for total MFM score.^15^ A lack of standardization and precision regarding how transfer capacity tasks were assessed in participants was reported from the studies.

#### Going up and down stairs

As shown in Supplementary Tables S1 Table 9 and S2 Table 9, 17 studies (31.5%) assessed going up and down stairs capacity. Distribution of COAs is presented in Figure 4D. When using a *PROM*, climbing stairs limitation was reported in 36.4%^32^ to 81.3%^30^ of patients in three studies, including one study where 68.0%^12^ of patients reported stairs limitations. When the limitation was present, it was frequently assessed using a *ClinRO* in 10 studies.^14,16,28,37–39,43,56,62,66^ From these, one retrospective study reported an incapacity to climb stairs independently in 76/302 patients (25.1%), with more than half of them aged younger than 75.^14^ Regarding *PerfO*, four studies used the same COA (10-step stair test) and reported an incapacity to execute the test in 18.8%^30^ to 20.9%^19,63^ of participants.^9,13,19,63^ Significant decline of 10-step stair test performance over a period of 20 months was reported in two studies.^19,63^ One study found significant difference between three age groups for the 10-step stair test performance at comfortable speed (during ascent and during descent) and at maximal speed (during ascent and during descent).^9^ In this same study using the 10-step stair test, percentage from reference values (only available for maximal speed) ranged from 45.1% to 85.1% (mean = 65.5%) during ascent and 54.7% to 84.4% (mean = 70.0%) during descent.^9^ Differences among studies regarding standardization of the 10-step stair test are reported, with a lack of precision regarding the testing conditions (use of handrails, walking aid and use of assistive devices).

## Discussion

The objective of this scoping review was to map muscle weakness, balance impairments and indoor mobility limitations documented in OPMD. More than half of the studies were case studies, which weakened the conclusions regarding the frequency and severity of muscle and balance impairments and indoor mobility limitations. Regarding COAs, unstandardized *ClinRO* was the most often used and only a few studies used *PerfO*, thus limiting comparison between studies and clear conclusion. In addition, validated patient-reported outcomes were completely absent for the concepts under study. Furthermore, the small number of prospective studies is alarming since this type of study design is key to clinical trial readiness. The results of this scoping review highlight the need for conducting larger prospective and longitudinal studies using standardized COAs to ensure data quality and support data pooling and/or comparison between studies. Nevertheless, some key findings emerged from this scoping review.

This scoping review highlighted that proximal upper and lower limb muscle weaknesses were more frequently assessed than distal and axial muscle weaknesses, regardless of the category of COA used. When using a *PerfO*, most studies used MMT rather than QMT. This is an important issue as several studies across different populations are reporting poor metrological properties of MMT including myotonic dystrophy type 1.^71–73^ This questions the validity of the findings in the different studies. For QMT, the dynamometer used along with the standardization of the method were different across studies, which limits the comparisons and for some the use of reference values. For example, the lever arm was taken into consideration in only one study,^9^ dampening the strength of the conclusions since comparison between individuals with different heights is not feasible. Therefore, considering that studies used different approaches to assess muscle strength and used different reference values, it is more complex to map impairment frequency and severity in OPMD from the current literature.

For lower limbs, knee extensors and hip flexors were assessed in all five studies using QMT, while the other muscle groups were sparsely assessed or not at all. However, cross-sectional studies assessing the pattern of muscle involvement in OPMD using MRI reported that knee flexors (semi-membranous and biceps femoris muscles),^33,35^ hip adductors (adductor magnus muscle),^33,62^ ankle plantar flexors (soleus and lateral gastrocnemius muscles)^33,35,62,74^ and ankle evertors (peroneus muscle)^35,74^ were the most impaired muscles according to the percentage of fatty infiltration, which is a biomarker of muscle deterioration.^33,35^ The adductor magnus muscle was also proposed to be a “sentinel” muscle to assess during clinical trials using MRI in OPMD^62^, but strength impairment related to adductor muscles has never been described in the literature previously.

The same observation is present for upper limbs, where shoulder abductors (deltoid muscle) were the only muscle group assessed in four studies using QMT, while the others were sparsely assessed or not at all. Again, cross-sectional studies assessing the pattern of muscle involvement in OPMD using MRI reported more prominent fatty infiltration of the shoulder protractors (serratus anterior),^33,62^ followed by shoulder internal rotator (subscapularis),^33^ while the rest of the shoulder muscles were spared.^33^

Regarding axial muscles, it was mostly assessed using unstandardized *ClinRO* and using MMT as a *PerfO*. Only one small cross-sectional study used QMT and showed no impairments regarding the neck flexors and extensors muscle groups.^13^ On the contrary, literature in OPMD showed some degree of fatty infiltrations in paraspinal cervical muscles using MRI in 30.9% of a large cohort.^33^

Considering the different protocols for muscle strength assessment using QMT, we cannot conclude from the studies reviewed if there is a difference between proximal and distal/axial weaknesses in terms of prevalence and severity using QMT assessment. In addition, due to the absence of consensus regarding the method to assess muscle strength using QMT, efforts should be made to standardize the assessment of muscle strength of all pertinent muscle groups (with the use of reference values) to assess the severity and progression of muscle weakness in neuromuscular disease, as suggested from MRI findings and from other diseases.^75–77^

Furthermore, discrepancies are noted between *PROM* and *PerfO* for the same category of muscle weakness. For example, hand weakness was not a reported symptom by the majority of participants in a qualitative study^12^ (considered as a *PROM* in this scoping review), while percentage of reference values for grip and pinch strength using a *PerfO* showed a mean percentage from reference values of 67.1% in a different study.^9^ This suggests a need to thoroughly explore the muscle weakness experience of patients in OPMD to fully capture the spectrum of the disease.

Static and dynamic balance were assessed in only seven studies, from which the same *PerfO* was used in four studies (Motor Function Measurement; MFM. Part D1 (standing and transfers tasks)).^15,19,35,63^ No specific balance assessment was performed in OPMD, which is surprising given the muscle impairment described, and the higher risk of falls related to aging. Furthermore, balance issues were reported in a qualitative study,^12^ which further supports the need to assess it.

Only five studies^9,13,15,19,63^ assessed specifically at least one component of indoor mobility according to definitions of Shumway-Cook et al.^11^ using a specific *PerfO*. Walking short distance was assessed using the 10mWT^9^ and the 6MWT,^15^ changing basic body position using the 30s-STS,^9^ and going up or down the stairs using the 10-step stair test.^9,13,19,63^ Only one study with a small sample size (n = 34) assessed all three indoor mobility concepts.^9^

Regarding short-distance walking limitations, only one study compared with reference values and found that comfortable speed was below 85% of reference values for participants more than 65 years old.^9^ This could suggest a more frequent limitation than reported in the literature. The same observation applies to changing basic body position assessed using the 30 s sit-to-stand, which showed performances as low as 50.3% of the reference values.^9^ As standing from sitting position requires hip extensors (gluteus maximus), knee flexors (biceps femoris) and knee extensors (rectus femoris, vastus medialis) muscle strength^11^ and balance to maintain the standing position, and considering that strength from the knee extensors had percentage from reference values at 74.0%,^9^ transfer capacity might be more frequently impaired than reported in the literature.

Use of stairs limitations were assessed using the same *PerfO* (10-step stair test) in four studies in OPMD,^9,13,19,63^ using different standardizations. The test did not show a significant difference between OPMD patients and control patients in a cross-sectional study,^13^ but deteriorated significantly over a 20-month period in longitudinal studies^19,63^ and showed limitations according to percentage from reference values in one study.^9^ Stair negotiation also mostly requires hip flexors and extensors and knee flexors and extensors muscle strength and involves dynamic balance since single limb stance is repeatedly required.

Mobility limitations, while present in OPMD according to this scoping review, showed no documented explanatory factors. Indeed, muscle strength is an important part of mobility, and hip flexors muscle weakness has been previously associated with the use of an assistive device in OPMD.^54^ Other variables like age, cognitive impairment, depressive symptoms, fear of falling, fatigue, and nutrition have shown an association with decreased mobility in older adults.^78–80^ All these factors should be further studied in OPMD. Given the late onset of OPMD, aging is a particularly important factor in this disease and should be taken into consideration in future studies. The results must be interpreted in the context of age-related expectations, ideally using reference or normative values when available, since other age-associated factors may influence the performance of individuals with OPMD. This is an essential step to develop rehabilitation interventions aiming to improve potential factors such as muscle strength, nutrition, and balance, as demonstrated in other neuromuscular diseases.^81–84^

Regarding COAs used in the different studies, a striking observation is the absence of standardized *PROM* both generic and disease-specific for the concept under study. However, this is a crucial and relevant category of COA, since it reflects the patient's perspective of a concept or subject and is now suggested by regulatory agencies to support clinical trial readiness. Only a few studies used nonspecific questionnaires, such as the Sickness Impact Profile^31^ which could include some individual questions around “ambulation”, “mobility”, capacity to rise from a chair or using stairs categories.^30–32,54^ In the literature, an OPMD-specific questionnaire exists only for dysphagia,^85^ but none was reported in our scoping review regarding muscle or balance impairments or mobility limitations. The use of PROM should be rapidly included in future studies to support a better understanding of the patient perspective.

## Conclusion

Key findings on muscle weakness, balance impairments and indoor mobility limitations in OPMD from this scoping review strongly suggest a need to 1) conduct large cross-sectional and longitudinal studies; 2) provide a complete portrait of muscle impairments; 3) document balance impairments; and 4) document indoor mobility limitations and its associated explanatory factors. In addition, the patient's voice is lacking as very few qualitative studies or use of PROM have been documented given the level of impairments and mobility limitations. These results will help guide clinicians and researchers in future studies to accelerate the development of drug therapy trials by using (or creating) adequate COAs in accordance with the FDA's guidelines for clinical trials.

**Abbreviations**
6MWT: 6-min walk test
10mWTc: 10-meter walk test comfortable speed
10mWTm: 10-meter walk test maximal speed
30s-STS: 30 s sit-to-stand test
BBS: Berg Balance Scale
BMI: Body-mass Index
ClinRO: Clinician-reported outcome measure
COA: Clinical outcome assessment
DM1: Myotonic Dystrophy type 1
HHD: Hand-held dynamometer
MFM: Motor Function Measurement
MMT: Manual muscle testing
MRC: Medical Research Council
MRI: Magnetic resonance imaging
N: Newtons
Nm: Newton-meters
OPMD: oculopharyngeal muscular dystrophy
PABPN1: Poly(A) Binding Protein Nuclear 1
PerfO: Performance outcome measure
PROM: Patient-reported outcome measure
QMT: Quantitative muscle testing
TUG: Timed Up & Go
SD: Standard Deviation
yo: years old

## Supplemental Material

sj-docx-1-jnd-10.1177_22143602251397052 - Supplemental material for What is known about muscle weakness, balance impairments and indoor mobility limitations in oculopharyngeal muscular dystrophy? A scoping review Supplemental material, sj-docx-1-jnd-10.1177_22143602251397052 for What is known about muscle weakness, balance impairments and indoor mobility limitations in oculopharyngeal muscular dystrophy? A scoping review by Nicolas Bélair, Cynthia Gagnon and Elise Duchesne in Journal of Neuromuscular Diseases

sj-docx-2-jnd-10.1177_22143602251397052 - Supplemental material for What is known about muscle weakness, balance impairments and indoor mobility limitations in oculopharyngeal muscular dystrophy? A scoping review Supplemental material, sj-docx-2-jnd-10.1177_22143602251397052 for What is known about muscle weakness, balance impairments and indoor mobility limitations in oculopharyngeal muscular dystrophy? A scoping review by Nicolas Bélair, Cynthia Gagnon and Elise Duchesne in Journal of Neuromuscular Diseases

sj-xlsx-3-jnd-10.1177_22143602251397052 - Supplemental material for What is known about muscle weakness, balance impairments and indoor mobility limitations in oculopharyngeal muscular dystrophy? A scoping review Supplemental material, sj-xlsx-3-jnd-10.1177_22143602251397052 for What is known about muscle weakness, balance impairments and indoor mobility limitations in oculopharyngeal muscular dystrophy? A scoping review by Nicolas Bélair, Cynthia Gagnon and Elise Duchesne in Journal of Neuromuscular Diseases

sj-docx-4-jnd-10.1177_22143602251397052 - Supplemental material for What is known about muscle weakness, balance impairments and indoor mobility limitations in oculopharyngeal muscular dystrophy? A scoping review Supplemental material, sj-docx-4-jnd-10.1177_22143602251397052 for What is known about muscle weakness, balance impairments and indoor mobility limitations in oculopharyngeal muscular dystrophy? A scoping review by Nicolas Bélair, Cynthia Gagnon and Elise Duchesne in Journal of Neuromuscular Diseases

## Appendices

### Appendix A: Complete search strategy

(“muscular dystrophy, oculopharyngeal"[MeSH Terms] OR (“muscular"[All Fields] AND “dystrophy"[All Fields] AND “oculopharyngeal"[All Fields]) OR “oculopharyngeal muscular dystrophy"[All Fields] OR (“oculopharyngeal"[All Fields] AND “muscular"[All Fields] AND “dystrophy"[All Fields]) OR (“Muscular Dystrophies"[MeSH Terms] AND “oculopharyngeal"[All Fields])) AND (((“motor"[All Fields] OR “motor s"[All Fields] OR “motoric"[All Fields] OR “motorically"[All Fields] OR “motorics"[All Fields] OR “motoring"[All Fields] OR “motorisation"[All Fields] OR “motorised"[All Fields] OR “motorization"[All Fields] OR “motorized"[All Fields] OR “motors"[All Fields]) AND “impair*"[All Fields]) OR (“muscle s"[All Fields] OR “muscles"[MeSH Terms] OR “muscles"[All Fields] OR “muscle"[All Fields]) OR “mobilit*"[All Fields] OR (“balance"[All Fields] OR “balanced"[All Fields] OR “balances"[All Fields] OR “balancing"[All Fields]) OR ((“functional"[All Fields] OR “functional s"[All Fields] OR “functionalities"[All Fields] OR “functionality"[All Fields] OR “functionalization"[All Fields] OR “functionalizations"[All Fields] OR “functionalize"[All Fields] OR “functionalized"[All Fields] OR “functionalizes"[All Fields] OR “functionalizing"[All Fields] OR “functionally"[All Fields] OR “functionals"[All Fields] OR “functioned"[All Fields] OR “functioning"[All Fields] OR “functionings"[All Fields] OR “functions"[All Fields] OR “physiology"[MeSH Subheading] OR “physiology"[All Fields] OR “function"[All Fields] OR “physiology"[MeSH Terms]) AND “capacit*"[All Fields])) AND (“human s"[All Fields] OR “humans"[MeSH Terms] OR “humans"[All Fields] OR “human"[All Fields])

### Appendix C: Final version of the extraction grid

**Table:**

Article details	Article 1	Article 2 …
Title		
Country		
Design		
Objectives		
Rationale		
Measurement tools		
Study eligibility and participants inclusion		
Population	Article 1	Article 2 …
Number of participants		
Age (mean), years		
Age (min., max.), years		
Symptoms or disease duration (mean and min-max), years		
Age and symptoms at examination (mean or median, number and %)		
Age at diagnosis (mean or median, number, min-max and range), years. PR = patient reported. MR = in Medical Record CR = clinicaly reported		
Age of first symptom onset (mean or median (Md), min-max and range), years. PR = patient reported. MR = in Medical Record CR = clinically reported		
Ethnicity		
Sex (n, %)		
Comorbidities (type, n, %)		
Inclusion and exclusion of participants		
Number of (GCN) expansion		
Other		
Motor impairments	Article 1	Article 2 …
Axial muscle weakness (neck, facial, oculomotor, trunk and abdominal muscles)		
Proximal lower limb muscle weakness (hip and knee)		
Proximal upper limb muscle weakness (shoulder, scapula and elbow)		
Upper and/or lower distal limb muscle weakness (wrist, ankle)		
Mobility limitations	Article 1	Article 2 …
Short distance walking (d4500)		
Changing basic body positions (d410)		
Going up and down stairs (d451)		
Other		
Comments		
