# Supplementary material for: What is known about muscle weakness, balance impairments and indoor mobility limitations in oculopharyngeal muscular dystrophy? A scoping review
Source: J Neuromuscul Dis. 2025 Dec 30:22143602251397052. Online ahead of print. doi: 10.1177/22143602251397052 (PMC13434899; doi:10.1177/22143602251397052)
Supplement: Supplementary file 1 [file sj-docx-1-jnd-10.1177_22143602251397052.docx]

**Supplementary Tables S1 (1 – 9)**

**Legend:**

- PROM: Patient-reported outcome measure
- ClinRO: Clinician-reported outcome measure
- PerfO: Performance outcome measure
- Pale grey: case studies, not accounted for prevalence range
- Underlined: case studies with large sample size, accounted for prevalence range

**S1 Table 1:** Studies reporting COA for proximal **lower proximal limb** muscle weakness.

|  | **PROM** | Interviews and questionnaires | **ClinRO** | Clinical observation | Patient file review | **PerfO** | MMT | QMT |
| --- | --- | --- | --- | --- | --- | --- | --- | --- |
| **Proximal lower limb muscle weakness** |  |  |  |  |  |  |  |  |
| **Case study** |  |  |  |  |  |  |  |  |
| Bilgen, C. et al. 2001 |  |  |  | x |  |  |  |  |
| Blumen, S. C. et al. 2013 |  |  |  | x |  |  | x |  |
| Bosch, E. P. et al. 1979 |  |  |  | x |  |  | x |  |
| Creel, G. B. et al. 1998 |  |  |  | x |  |  |  |  |
| Fardeau, M. et al. 1997 |  |  |  | x |  |  |  |  |
| Fukuhara, N. et al. 1982 |  |  |  | x |  |  |  |  |
| Hill, M. et al. 2001 |  |  |  | x |  |  |  |  |
| Kuo, H. C. et al. 2009 |  |  |  | x |  |  |  |  |
| Medici, M. et al. 1997 |  |  |  | x |  |  |  |  |
| Mihaylova, V. et al. 2008 |  |  |  | x |  |  | x |  |
| Müller, T. et al. 2001 |  |  |  | x |  |  |  |  |
| Nagashima, T. et al. 2000 |  |  |  | x |  |  |  |  |
| Nishii, Y. S et al. 2021 |  |  |  | x |  |  | x |  |
| Piccolo, G. et al. 2011 |  |  |  | x |  |  | x |  |
| Porschke, H. et al. 1997 |  |  |  | x |  |  |  |  |
| Robinson, D. O. et al. 2011 |  |  |  | x |  |  |  |  |
| Rüegg, S. et al. 2005 |  |  |  | x |  |  |  |  |
| Saini, M. et al. 2018 |  |  |  |  |  |  | x |  |
| Salvesen, R. et al. 1996 |  |  |  | x |  |  |  |  |
| Schmitt, H. P. et al. 1981 |  |  |  | x |  |  |  |  |
| Schober, B. G. et al. 2001 |  |  |  | x |  |  | x |  |
| Shan, J. et al. 2014 |  |  |  | x |  |  |  |  |
| Teh, B. T. et al. 1997 |  |  |  | x |  |  |  |  |
| Tremolizzo, L. et al. 2007 |  |  |  | x |  |  |  |  |
| Uyama, E. et al. 1996 |  |  |  |  |  |  | x |  |
| Uyama, E. et al. 1997 |  |  |  |  |  |  | x |  |
| Vita, G. et al. 1983 |  |  |  | x |  |  | x |  |
| **Prospective study** |  |  |  |  |  |  |  |  |
| Kroon et al. 2021 |  |  |  |  |  |  | x | x |
| Kroon et al. 2022 |  |  |  |  |  |  | x | x |
| **Cross-sectional study** |  |  |  |  |  |  |  |  |
| Alonsò-Jimenez, A. et al. 2019 |  |  |  | x |  |  |  |  |
| Blumen, S. C. et al. 1993 |  |  |  | x |  |  |  |  |
| Brisson, J-D. et al. 2023 |  |  |  |  |  |  |  | x |
| Fischmann, A. et al. 2011 |  |  |  | x |  |  |  |  |
| Goyal, N. A. et al. 2019 |  |  |  |  |  |  | x |  |
| Melkus, G. et al. 2023 |  |  |  |  |  |  | x |  |
| Tremblay-Tymczuk, S. et al. 1992 |  | x |  |  |  |  |  |  |
| van der Sluijs, B. M. et al. 2003 |  | x |  |  |  |  | x |  |
| van der Sluijs, B. M. et al. 2016a |  | x |  |  |  |  |  |  |
| van der Sluijs, B. M. et al. 2017 |  | x |  |  |  |  | x | x |
| van der Sluijs, B. M. et al. 2016b |  |  |  |  |  |  | x |  |
| Witting, N. et al. 2014 |  |  |  |  |  |  |  | x |
| Youssof, S. 2016 |  |  |  | x |  |  |  |  |
| **Retrospective study** |  |  |  |  |  |  |  |  |
| Agarwal, P. K. et al. 2012 |  |  |  |  | x |  |  |  |
| Alonsò-Perez, J. et al. 2022 |  |  |  |  | x |  | x |  |
| Becher, M. W. et al. 2001 |  |  |  |  | x |  |  |  |
| Blumen, S. C. et al. 1997 |  |  |  |  | x |  |  |  |
| Brisson, J-D. et al. 2020 |  |  |  |  | x |  |  |  |
| Jones, L. et al. 2010 |  |  |  |  | x |  |  |  |
| Youssof, S. et al. 2015 |  |  |  |  | x |  | x |  |
| **Qualitative study** |  |  |  |  |  |  |  |  |
| Kurtz, N. S. et al. 2019 |  | x |  |  |  |  |  |  |
| **Total number of studies: n = 50** |  | 5 |  | 28 | 7 |  | 19 | 5 |

**S1 Table 2**: Studies reporting COA for **distal lower limb** muscle weakness

|  | **PROM** | Interviews and questionnaires | **ClinRO** | Clinical observation | Patient file review | **PerfO** | MMT | QMT |
| --- | --- | --- | --- | --- | --- | --- | --- | --- |
| **Distal lower limb muscle weakness** |  |  |  |  |  |  |  |  |
| **Case study** |  |  |  |  |  |  |  |  |
| Bilgen, C. et al. 2001 |  |  |  | x |  |  |  |  |
| Blumen, S. C. et al. 2013 |  |  |  | x |  |  | x |  |
| Bosch, E. P. et al. 1979 |  |  |  | x |  |  | x |  |
| Creel, G. B. et al. 1998 |  |  |  | x |  |  |  |  |
| Fardeau, M. et al. 1997 |  |  |  | x |  |  |  |  |
| Fukuhara, N. et al. 1982 |  |  |  | x |  |  |  |  |
| Mihaylova, V. et al. 2008 |  |  |  | x |  |  | x |  |
| Nishii, Y. S et al. 2021 |  |  |  | x |  |  | x |  |
| Piccolo, G. et al. 2011 |  |  |  | x |  |  |  |  |
| Ruëgg et al. 2005 |  |  |  | x |  |  |  |  |
| Salvesen, R. et al. 1996 |  |  |  | x |  |  |  |  |
| Schmitt, H. P. et al. 1981 |  |  |  | x |  |  |  |  |
| Schober, B. G. et al. 2001 |  |  |  | x |  |  | x |  |
| Shan, J. et al. 2014 |  |  |  | x |  |  |  |  |
| Uyama, E. et al. 1996 |  |  |  |  |  |  | x |  |
| Uyama, E. et al. 1997 |  |  |  |  |  |  | x |  |
| Vita, G. et al. 1983 |  |  |  | x |  |  | x |  |
| **Prospective study** |  |  |  |  |  |  |  |  |
| Kroon et al. 2021 |  |  |  |  |  |  | x |  |
| Kroon et al. 2022 |  |  |  |  |  |  | x |  |
| **Cross-sectional study** |  |  |  |  |  |  |  |  |
| Alonsò-Jimenez, A. et al. 2019 |  |  |  | x |  |  |  |  |
| Blumen, S. C. et al. 1993 |  |  |  | x |  |  |  |  |
| Goyal, N. A. et al. 2019 |  |  |  |  |  |  | x |  |
| van der Sluijs, B. M. et al. 2003 |  | x |  |  |  |  | x |  |
| van der Sluijs, B. M. et al. 2017 |  | x |  |  |  |  | x |  |
| van der Sluijs, B. M. et al. 2016b |  |  |  | x |  |  | x |  |
| Witting, N. et al. 2014 |  |  |  |  |  |  |  | x |
| **Retrospective study** |  |  |  |  |  |  |  |  |
| Alonsò-Perez, J. et al. 2022 |  |  |  |  | x |  | x |  |
| Brisson, J-D. et al. 2020 |  |  |  |  | x |  | x |  |
| Jones, L. et al. 2010 |  |  |  |  | x |  |  |  |
| Youssof, S. et al. 2015 |  |  |  |  | x |  | x |  |
| **Total number of studies: n = 30** |  | 2 |  | 18 | 4 |  | 18 | 1 |

**S1 Table 3:** Studies reporting COA for **proximal upper limb** muscle weakness

|  | **PROM** | Interviews and questionnaires | **ClinRO** | Clinical observation | Patient file review | **PerfO** | MMT | QMT | Other test |
| --- | --- | --- | --- | --- | --- | --- | --- | --- | --- |
| **Proximal upper limb muscle weakness** |  |  |  |  |  |  |  |  |  |
| **Case study** |  |  |  |  |  |  |  |  |  |
| Bilgen, C. et al. 2001 |  |  |  | x |  |  |  |  |  |
| Blumen, S. C. et al. 2013 |  |  |  | x |  |  | x |  |  |
| Bosch, E. P. et al. 1979 |  |  |  | x |  |  | x |  |  |
| Creel, G. B. et al. 1998 |  |  |  | x |  |  |  |  |  |
| Fardeau, M. et al. 1997 |  |  |  | x |  |  |  |  |  |
| Fukuhara, N. et al. 1982 |  |  |  | x |  |  |  |  |  |
| Hill, M. E. et al. 2001 |  |  |  | x |  |  |  |  |  |
| Kuo, H. C. et al. 2009 |  |  |  | x |  |  |  |  |  |
| Medici, M. et al. 1997 |  |  |  | x |  |  |  |  |  |
| Mihaylova, V. et al. 2008 |  |  |  | x |  |  | x |  |  |
| Müller, T. et al. 2001 |  |  |  | x |  |  |  |  |  |
| Munitiz, V. et al. 2003 |  |  |  | x |  |  |  |  |  |
| Nagashima, T. et al. 2000 |  |  |  | x |  |  |  |  |  |
| Nishii, Y. S et al. 2021 |  |  |  | x |  |  | x |  |  |
| Piccolo, G. et al. 2011 |  |  |  | x |  |  |  |  |  |
| Porschke, H. et al. 1997 |  |  |  | x |  |  |  |  |  |
| Robinson, D. O. et al. 2011 |  |  |  | x |  |  |  |  |  |
| Rüegg, S. et al. 2005 |  |  |  | x |  |  |  |  |  |
| Saini, M. et al. 2018 |  |  |  |  |  |  | x |  |  |
| Salvesen, R. et al. 1996 |  |  |  | x |  |  |  |  |  |
| Schmitt, H. P. et al. 1981 |  |  |  | x |  |  |  |  |  |
| Schober, B. G. et al. 2001 |  |  |  | x |  |  | x |  |  |
| Shan, J. et al. 2014 |  |  |  | x |  |  |  |  |  |
| Teh, B. T. et al. 1997 |  |  |  | x |  |  |  |  |  |
| Tremolizzo, L. et al. 2007 |  |  |  | x |  |  |  |  |  |
| Uyama, E. et al. 1996 |  |  |  |  |  |  | x |  |  |
| Uyama, E. et al. 1997 |  |  |  |  |  |  | x |  |  |
| Vita, G. et al. 1983 |  |  |  | x |  |  | x |  |  |
| **Prospective study** |  |  |  |  |  |  |  |  |  |
| Kroon et al. 2021 |  |  |  |  |  |  | x | x |  |
| Kroon et al. 2022 |  |  |  |  |  |  | x | x |  |
| **Cross-sectional study** |  |  |  |  |  |  |  |  |  |
| Alonsò-Jimenez, A. et al. 2019 |  |  |  | x |  |  |  |  |  |
| Blumen, S. C. et al. 1993 |  |  |  | x |  |  |  |  |  |
| Brisson, J-D. et al. 2023 |  |  |  |  |  |  |  | x |  |
| Goyal, N. A. et al. 2019 |  |  |  | x |  |  | x |  |  |
| Melkus, G. et al. 2023 |  |  |  |  |  |  | x |  |  |
| Tremblay-Tymczuk, S. et al. 1992 |  | x |  |  |  |  |  |  | x |
| van der Sluijs, B. M. et al. 2003 |  | x |  |  |  |  | x |  |  |
| van der Sluijs, B. M. et al. 2016a |  | x |  |  |  |  |  |  |  |
| van der Sluijs, B. M. et al. 2017 |  | x |  |  |  |  | x |  |  |
| van der Sluijs, B. M. et al. 2016b |  |  |  |  |  |  | x |  |  |
| Witting, N. et al. 2014 |  |  |  |  |  |  |  | x |  |
| Youssof, S. 2016 |  |  |  | x |  |  |  |  |  |
| **Retrospective study** |  |  |  |  |  |  |  |  |  |
| Agarwal, P. K. et al. 2012 |  |  |  |  | x |  |  |  |  |
| Alonsò-Perez, J. et al. 2022 |  |  |  |  | x |  | x |  |  |
| Becher, M. W. et al. 2001 |  |  |  |  | x |  |  |  |  |
| Blumen, S. C. et al. 1997 |  |  |  |  | x |  |  |  |  |
| Brisson, J-D. et al. 2020 |  |  |  |  | x |  | x |  |  |
| Jones, L. et al. 2010 |  |  |  |  | x |  |  |  |  |
| Youssof, S. et al. 2015 |  |  |  |  | x |  | x |  |  |
| **Qualitative study** |  |  |  |  |  |  |  |  |  |
| Kurtz, N. S. et al. 2019 |  | x |  |  |  |  |  |  |  |
| **Total number of articles: n = 50** |  | 5 |  | 29 | 7 |  | 19 | 4 | 1 |

**S1 Table 4**: Studies reporting COA for **distal upper limb** muscle weakness.

|  | **PROM** | Interview and questionnaires | **ClinRO** | Clinical observation | Patient file review | **PerfO** | MMT | QMT |
| --- | --- | --- | --- | --- | --- | --- | --- | --- |
| **Distal upper limb muscle weakness** |  |  |  |  |  |  |  |  |
| **Case study** |  |  |  |  |  |  |  |  |
| Bilgen, C. et al. 2001 |  |  |  | x |  |  |  |  |
| Blumen, S. C. et al. 2013 |  |  |  | x |  |  | x |  |
| Bosch, E. P. et al. 1979 |  |  |  | x |  |  | x |  |
| Creel, G. B. et al. 1998 |  |  |  | x |  |  |  |  |
| Fardeau, M. et al. 1997 |  |  |  | x |  |  |  |  |
| Fukuhara, N. et al. 1982 |  |  |  | x |  |  |  |  |
| Mihaylova, V. et al. 2008 |  |  |  | x |  |  | x |  |
| Nishii, Y. S et al. 2021 |  |  |  | x |  |  | x |  |
| Piccolo, G. et al. 2011 |  |  |  | x |  |  |  |  |
| Ruëgg et al. 2005 |  |  |  | x |  |  |  |  |
| Salvesen, R. et al. 1996 |  |  |  | x |  |  |  |  |
| Schmitt, H. P. et al. 1981 |  |  |  | x |  |  |  |  |
| Schober, B. G. et al. 2001 |  |  |  | x |  |  | x |  |
| Shan, J. et al. 2014 |  |  |  | x |  |  |  |  |
| Uyama, E. et al. 1996 |  |  |  | x |  |  | x |  |
| Uyama, E. et al. 1997 |  |  |  | x |  |  | x |  |
| Vita, G. et al. 1983 |  |  |  | x |  |  | x |  |
| **Prospective study** |  |  |  |  |  |  |  |  |
| Kroon et al. 2021 |  |  |  |  |  |  | x |  |
| Kroon et al. 2022 |  |  |  |  |  |  | x |  |
| **Cross-sectional study** |  |  |  |  |  |  |  |  |
| Alonsò-Jimenez, A. et al. 2019 |  |  |  | x |  |  |  |  |
| Blumen, S. C. et al. 1993 |  |  |  | x |  |  |  |  |
| Brisson, J-D. et al. 2023 |  |  |  |  |  |  |  | x |
| Goyal, N. A. et al. 2019 |  |  |  |  |  |  | x |  |
| Tremblay-Tymczuk, S. et al. 1992 |  |  |  |  |  |  |  | x |
| van der Sluijs, B. M. et al. 2003 |  | x |  |  |  |  | x |  |
| van der Sluijs, B. M. et al. 2017 |  | x |  |  |  |  | x |  |
| van der Sluijs, B. M. et al. 2016b |  |  |  |  |  |  | x |  |
| Witting, N. et al. 2014 |  |  |  |  |  |  |  | x |
| **Retrospective study** |  |  |  |  |  |  |  |  |
| Alonsò-Perez, J. et al. 2022 |  |  |  |  | x |  | x |  |
| Brisson, J-D. et al. 2020 |  |  |  |  | x |  | x |  |
| Jones, L. et al. 2010 |  |  |  |  | x |  |  |  |
| Youssof, S. et al. 2015 |  |  |  |  | x |  | x |  |
| **Qualitative study** |  |  |  |  |  |  |  |  |
| Kurtz, N. S. et al. 2019 |  | x |  |  |  |  |  |  |
| **Total number of articles: n = 33** |  | 3 |  | 19 | 4 |  | 17 | 3 |

**S1 Table 5:** Studies reporting COA for **axial** muscle weakness

|  | **PROM** | Interviews and questionnaires | **ClinRO** | Clinical observation | Patient file review | **PerfO** | MMT | QMT | Other tests |
| --- | --- | --- | --- | --- | --- | --- | --- | --- | --- |
| **Axial muscle weakness** |  |  |  |  |  |  |  |  |  |
| **Case study** |  |  |  |  |  |  |  |  |  |
| Bilgen, C. et al. 2001 |  |  |  | x |  |  |  |  |  |
| Blumen, S. C. et al. 2013 |  |  |  | x |  |  | x |  |  |
| Bosch, E. P. et al. 1979 |  |  |  | x |  |  |  |  |  |
| Creel, G. B. et al. 1998 |  |  |  | x |  |  |  |  |  |
| Dobrowski, G. M. et al. 1986 |  |  |  | x |  |  |  |  |  |
| Eustace, S. et al. 1982 |  |  |  | x |  |  |  |  |  |
| Fardeau, M. et al. 1997 |  |  |  | x |  |  |  |  |  |
| Fukuhara, N. et al. 1982 |  |  |  | x |  |  |  |  |  |
| Kuo, H. C. et al. 2009 |  |  |  | x |  |  |  |  |  |
| Medici, M. et al. 1997 |  |  |  | x |  |  |  |  |  |
| Mihaylova, V. et al. 2008 |  |  |  | x |  |  |  |  |  |
| Munitiz, V. et al. 2003 |  |  |  | x |  |  |  |  |  |
| Müller, T. et al. 2001 |  |  |  | x |  |  |  |  |  |
| Nagashima, T. et al. 2000 |  |  |  | x |  |  |  |  |  |
| Nishii, Y. S et al. 2021 |  |  |  | x |  |  | x |  |  |
| Piccolo, G. et al. 2011 |  |  |  | x |  |  |  |  |  |
| Porschke, H. et al. 1997 |  |  |  | x |  |  |  |  |  |
| Robinson, D. O. et al. 2011 |  |  |  | x |  |  |  |  |  |
| Rüegg, S. et al. 2005 |  |  |  | x |  |  |  |  |  |
| Saini, M. et al. 2018 |  |  |  | x |  |  | x |  |  |
| Salvesen, R. et al. 1996 |  |  |  | x |  |  |  |  |  |
| Schmitt, H. P. et al. 1981 |  |  |  | x |  |  |  |  |  |
| Schober, B. G. et al. 2001 |  |  |  | x |  |  |  |  |  |
| Shan, J. et al. 2014 |  |  |  | x |  |  |  |  |  |
| Teh, B. T. et al. 1997 |  |  |  | x |  |  |  |  |  |
| Tremolizzo, L. et al. 2007 |  |  |  | x |  |  |  |  |  |
| Uyama, E. et al. 1996 |  |  |  | x |  |  | x |  |  |
| Uyama, E. et al. 1997 |  |  |  | x |  |  | x |  |  |
| Vita, G. et al. 1983 |  |  |  | x |  |  |  |  |  |
| **Prospective study** |  |  |  |  |  |  |  |  |  |
| Kroon et al. 2021 |  |  |  |  |  |  | x |  |  |
| Kroon et al. 2022 |  |  |  |  |  |  | x |  |  |
| **Cross-sectional study** |  |  |  |  |  |  |  |  |  |
| Alonsò-Jimenez, A. et al. 2019 |  |  |  | x |  |  |  |  |  |
| Blumen, S. C. et al. 1993 |  |  |  | x |  |  |  |  |  |
| Fischmann et al. 2011 |  |  |  | x |  |  |  |  |  |
| Goyal, N. A. et al. 2019 |  |  |  | x |  |  | x |  |  |
| Tremblay-Tymczuk, S. et al. 1992 |  |  |  | x |  |  |  |  | x |
| van der Sluijs, B. M. et al. 2003 |  | x |  | x |  |  | x |  |  |
| van der Sluijs, B. M. et al. 2016a |  | x |  | x |  |  |  |  |  |
| van der Sluijs, B. M. et al. 2017 |  | x |  | x |  |  | x |  |  |
| van der Sluijs, B. M. et al. 2016b |  | x |  | x |  |  | x |  |  |
| Witting, N. et al. 2014 |  |  |  | x |  |  | x | x |  |
| Youssof, S. 2016 |  |  |  | x |  |  |  |  |  |
| **Retrospective study** |  |  |  |  |  |  |  |  |  |
| Agarwal, P. K. et al. 2012 |  |  |  |  | x |  |  |  |  |
| Alonsò-Perez, J. et al. 2022 |  |  |  |  | x |  | x |  |  |
| Becher, M. W. et al. 2001 |  |  |  |  | x |  |  |  |  |
| Blumen, S. C. et al. 1997 |  |  |  |  | x |  |  |  |  |
| Brisson, J-D. et al. 2020 |  |  |  |  | x |  |  |  |  |
| Jones, L. et al. 2010 |  |  |  |  | x |  |  |  |  |
| Youssof, S. et al. 2015 |  |  |  |  | x |  | x |  |  |
| **Qualitative study** |  |  |  |  |  |  |  |  |  |
| Kurtz, N. S. et al. 2019 |  | x |  |  |  |  |  |  |  |
| **Total number of articles (ptosis included): n = 50** |  |  |  | 40 | 7 |  |  |  |  |
| **Total number of articles (without ptosis): n = 32** |  | 5 |  | 16 | 1 |  | 14 | 1 | 1 |

**S1 Table 6:** Studies reporting COA for **balance impairment**

|  | **PROM** | Interviews and questionnaires | **ClinRO** | Clinical observation | **PerfO** | Other tests |
| --- | --- | --- | --- | --- | --- | --- |
| **Balance impairement** |  |  |  |  |  |  |
| **Case study** |  |  |  |  |  |  |
| Schmitt, H. P. et al. 1981 |  |  |  | x |  |  |
| Schober, R. et al. 2001 |  |  |  | x |  |  |
| **Prospective study** |  |  |  |  |  |  |
| Kroon et al. 2021 |  |  |  |  |  | x |
| Kroon et al. 2022 |  |  |  |  |  | x |
| **Cross-sectional study** |  |  |  |  |  |  |
| Fischmann, A. et al. 2011 |  |  |  |  |  | x |
| van der Sluijs, B. M. et al. 2017 |  |  |  |  |  | x |
| **Qualitative study** |  |  |  |  |  |  |
| Kurtz, N. S. et al. 2019 |  | x |  |  |  |  |
| **Total number of studies: n = 7** |  | 1 |  | 2 |  | 4 |

**S1 Table 7:** Studies reporting COA for **walking short distances (d4500) limitations**.

|  | **PROM** | Interviews and questionnaires | **ClinRO** | Clinical observation | Use of walking aid | Patient file review | **PerfO** | Walk test | Other tests |
| --- | --- | --- | --- | --- | --- | --- | --- | --- | --- |
| **Walking short distances (d4500)** |  |  |  |  |  |  |  |  |  |
| **Case study** |  |  |  |  |  |  |  |  |  |
| Blumen, S. C. et al. 2013 |  |  |  | x |  |  |  |  |  |
| Bosch, E. P. et al. 1979 |  |  |  | x |  |  |  |  |  |
| Fukuhara, N. et al. 1982 |  |  |  | x |  |  |  |  |  |
| Kuo, H. C. et al. 2009 |  |  |  | x |  |  |  |  |  |
| Müller, T. et al. 2001 |  |  |  | x |  |  |  |  |  |
| Nishii, Y. S. et al. 2021 |  |  |  | x |  |  |  |  |  |
| Porschke, H. et al. 1997 |  |  |  | x |  |  |  |  |  |
| Robinson, D. O. et al. 2011 |  |  |  | x |  |  |  |  |  |
| Ruëgg, S. et al. 2005 |  |  |  | x |  |  |  |  |  |
| Saini, M. et al. 2018 |  |  |  | x |  |  |  |  |  |
| Salvesen, R. et al. 1996 |  |  |  | x | x |  |  |  |  |
| Schmitt, H. P. et al. 1981 |  |  |  | x |  |  |  |  |  |
| Schober, B. G. et al. 2001 |  |  |  | x |  |  |  |  |  |
| Shan, J. et al. 2014 |  |  |  | x |  |  |  |  |  |
| Vita, G. et al. 1983 |  |  |  | x |  |  |  |  |  |
| **Prospective study** |  |  |  |  |  |  |  |  |  |
| Kroon et al. 2021 |  |  |  |  |  |  |  |  | x |
| Kroon et al. 2022 |  |  |  |  |  |  |  |  | x |
| **Cross-sectional study** |  |  |  |  |  |  |  |  |  |
| Alonsò-Jimenez, A. et al. 2019 |  |  |  |  | x |  |  |  |  |
| Blumen, S. C. et al. 1993 |  |  |  | x |  |  |  |  |  |
| Brisson, J-D. et al. 2023 |  |  |  |  |  |  |  | x |  |
| Fischmann et al. 2011 |  |  |  |  |  |  |  |  | x |
| Melkus, G. et al. 2023 8 |  |  |  |  | x |  |  |  |  |
| Tremblay-Tymczuk, S. et al. 1992 |  | x |  | x | x |  |  |  |  |
| van der Sluijs, B. M. et al. 2003 |  | x |  |  |  |  |  |  |  |
| van der Sluijs, B. M. et al. 2016a |  | x |  |  |  |  |  |  |  |
| van der Sluijs, B. M. et al. 2017 |  | x |  | x |  |  |  | x | x |
| Witting, N. et al. 2014 |  |  |  |  | x |  |  |  |  |
| Youssof, S. et al. 2016 |  | x |  |  | x |  |  |  |  |
| **Retrospective study** |  |  |  |  |  |  |  |  |  |
| Alonsò-Perez, J. et al. 2022 |  |  |  | x | x | x |  |  |  |
| Becher, M. W. et al. 2001 |  |  |  | x |  | x |  |  |  |
| Brisson, J-D. et al. 2020 |  |  |  |  | x | x |  |  |  |
| Wallace, B. et al. 2021 |  |  |  |  | x | x |  |  |  |
| Youssof, S. et al. 2015 |  |  |  |  | x | x |  |  |  |
| **Qualitative study** |  |  |  |  |  |  |  |  |  |
| Kurtz, N. S. et al. 2019 |  | x |  |  | x |  |  |  |  |
| **Total number of articles: n = 34** |  | 6 |  | 20 | 11 | 5 |  | 2 | 4 |

**S1 Table 8:** Studies reporting COA for **changing basic body positions (d410)** limitations.

|  | **PROM** | Interviews and questionnaires | **ClinRO** | Clinical observation | Patient file review | **PerfO** | Transfer test | Other tests |
| --- | --- | --- | --- | --- | --- | --- | --- | --- |
| **Changing basic body position (d410)** |  |  |  |  |  |  |  |  |
| **Case study** |  |  |  |  |  |  |  |  |
| Bosch, E. P. et al. 1979 |  |  |  | x |  |  |  |  |
| Fukuhara, N. et al. 1982 |  |  |  | x |  |  |  |  |
| Müller, T. et al. 2001 |  |  |  | x |  |  |  |  |
| Salvesen, R. et al. 1996 |  |  |  | x |  |  |  |  |
| Schmitt, H. P. et al. 1981 |  |  |  | x |  |  |  |  |
| Vita, G. et al. 1983 |  |  |  | x |  |  |  |  |
| **Prospective study** |  |  |  |  |  |  |  |  |
| Kroon et al. 2021 |  |  |  |  |  |  |  | x |
| Kroon et al. 2022 |  |  |  |  |  |  |  | x |
| **Cross-sectional study** |  |  |  |  |  |  |  |  |
| Blumen, S. C. et al. 1993 |  |  |  | x |  |  |  |  |
| Brisson, J-D. et al. 2023 |  |  |  |  |  |  | x |  |
| Fischmann et al. 2011 |  |  |  |  |  |  |  | x |
| Tremblay-Tymczuk, S. et al. 1992 |  | x |  | x |  |  |  |  |
| van der Sluijs, B. M. et al. 2003 |  | x |  | x |  |  |  |  |
| van der Sluijs, B. M. et al. 2017 |  |  |  |  |  |  |  | x |
| **Retrospective study** |  |  |  |  |  |  |  |  |
| Brisson, J-D. et al. 2020 |  |  |  |  | x |  |  |  |
| **Qualitative study** |  |  |  |  |  |  |  |  |
| Kurtz, N. S. et al. 2019 |  | x |  |  |  |  |  |  |
| **Total number of articles: n = 16** |  | 3 |  | 9 | 1 |  | 1 | 4 |

**S1 Table 9:** Studies reporting COA for **going up and down stairs (d451) limitations**.

|  | **PROM** | Interviews and questionnaires | **ClinRO** | Clinical observation | Patient file review | **PerfO** | Step test |
| --- | --- | --- | --- | --- | --- | --- | --- |
| **Going up and down stairs (d451)** |  |  |  |  |  |  |  |
| **Case study** |  |  |  |  |  |  |  |
| Blumen, S. C. et al. 2013 |  |  |  | x |  |  |  |
| Bosch, E. P. et al. 1979 |  |  |  | x |  |  |  |
| Fukuhara, N. et al. 1982 |  |  |  | x |  |  |  |
| Kuo, H. C. et al. 2009 |  |  |  | x |  |  |  |
| Müller, T. et al. 2001 |  |  |  | x |  |  |  |
| Nishii, Y. S. et al. 2021 |  |  |  | x |  |  |  |
| Porschke, H. et al. 1997 |  |  |  | x |  |  |  |
| Salvesen, R. et al. 1996 |  |  |  | x |  |  |  |
| Vita, G. et al. 1983 |  |  |  | x |  |  |  |
| **Prospective study** |  |  |  |  |  |  |  |
| Kroon et al. 2021 |  |  |  |  |  |  | x |
| Kroon et al. 2022 |  |  |  |  |  |  | x |
| **Cross-sectional study** |  |  |  |  |  |  |  |
| Brisson, J-D. et al. 2023 |  |  |  |  |  |  | x |
| Tremblay-Tymczuk, S. et al. 1992 |  | x |  |  |  |  |  |
| van der Sluijs, B. M. et al. 2003 |  | x |  |  |  |  |  |
| Witting, N. et al. 2014 |  |  |  |  |  |  | x |
| **Retrospective study** |  |  |  |  |  |  |  |
| Brisson, J-D. et al. 2020 |  |  |  |  | x |  |  |
| **Qualitative study** |  |  |  |  |  |  |  |
| Kurtz, N. S. et al. 2019 |  | x |  |  |  |  |  |
| **Total number of articles: n = 17** |  | 3 |  | 9 | 1 |  | 4 |
