# Supplementary material for: What is known about muscle weakness, balance impairments and indoor mobility limitations in oculopharyngeal muscular dystrophy? A scoping review
Source: J Neuromuscul Dis. 2025 Dec 30:22143602251397052. Online ahead of print. doi: 10.1177/22143602251397052 (PMC13434899; doi:10.1177/22143602251397052)
Supplement: Supplementary file 2 [file sj-docx-2-jnd-10.1177_22143602251397052.docx]

**Supplementary Tables S2 (1 – 9)**

**Legend:**

- COA: Clinical outcome assessment (category)
- PROM: Patient-reported outcome measure
- ClinRO: Clinician-reported outcome measure
- PerfO: Performance outcome measure
- yo: years old
- Pale grey: case studies, taken into consideration for the number of studies, but not for the prevalence
- Underlined: case studies with large sample size
- **Green bold text:** extreme values reported for prevalence range

**S2 Table 1**: Key findings of COA’s reporting **proximal lower limb** muscle weakness.

| **50 studies** | **COA** | **Method** | **Findings** |
| --- | --- | --- | --- |
| **PROM:** 5 different studies | | | |
| **Interviews**  (n = 1) | **PROM:** Semi-structured interview (n = 1) | « Framework »  technic | Proximal lower limb weakness reported from 19/25 (76.0%) patients. Small sample size. No precision regarding muscle groups or movements impaired (Kurtz et al. 2019). |
| **Questionnaire** (n = 4) | **PROM**: Homemade socio- demographic questionnaire (n = 4) | Presence of weakness? Duration? Use of a walking aid or wheelchair? | Proximal lower limb weakness in **6/14 (42.9%) to 20/22 (90.9%)** patients (Tremblay-Tymczuk et al. 1992, Van Der Sluijs et al. 2003, van der Sluijs et al. 2016a, van der Sluijs et al. 2017). Lower and upper proximal limb weakness in combination for 77.2% of patient sample in one study. Early onset of symptoms in 18 patients in their fifties (mean: 57 yo. Range: 45 – 69 yo). No precision on muscle groups or movements impaired. Binomial (yes/no) clinical outcome. Small to moderate sample size in the studies. |
| **ClinRO:** 35 different studies | | | |
| **Clinical observation**  (n = 28) | **ClinRO** : Physical examination of muscle strength (n = 28) | Neurological examination of muscle strength and/or presence of atrophy | Proximal lower limb weakness is more frequently reported alone than in combination with upper proximal limb weakness, symmetrical > asymmetrical. Studies designs are as follows: case-control > cross-sectional > retrospective > prospective. Small to moderate sample sizes. Severe to mild weakness reported in **3/31 (9.7%)** to 75/89 (84.3%) of patients (excluding case studies) (Alonso-Jimenez et al. 2019, Blumen et al. 1993, Fardeau et al. 1997, Fischmann et al. 2011, Hill et al. 2001, Medici et al. 1997, Porschke et al. 1997, Shan et al. 2014, Youssof 2016).  **Author’s last name, year: prevalence (%):** -Alonso-Jimenez et al 2018: 82/168 (48.8%)  -Bilgen et al. 2001: case study, 3/4 (75.0%) -Blumen et al. 1993: 21/79 (27.0%)  -Blumen et al. 2013: case study, 3/5 (60.0%)  -Bosch et al. 1979: case study, 1/1 (100.0%) -Creel et al. 1998: case study, 1/2 (50.0%) generalized limb weakness  -Fardeau et al. 1997: 15/29 (51.7%)  -Fischmann et al. 2011 : 5/8 (62.5%) -Fukuhara et al. 1982: case study, 2/2 (100.0%)  **-Hill et al. 2001: 3/31 (9.7%)** -Kuo et al. 2009: case study, 1/10 (10.0%) -Medici et al. 1997: 22/65 (33.9%)  -Mihaylova et al. 2008: case study, 1/4 (25.0%) -Muller et al. 2001 : 9/16 (56.3%)  -Nagashima et al. 2000: case study, 4/4 (100.0%)  -Nishii et al. 2021: case study, 1/1 (100.0%) -Piccolo et al. 2011: case study, 0/1 (0.0%) -Porschke et al. 1997: NR, only stating that some patients had proximal lower limb paresis and atrophy  -Robinson et al. 2011: case study, 1/2 (50.0%)  -Ruegg et al. 2005: illustrative case, no weakness (muscle strength normal). Prevalence of proximal weakness in other studies reported in the article:   - New Mexico: 32/49 (65.0%)   - Israel: 24/112 (20.0%)   - Quebec: 52/72 (71.0%)  - France: 15/29 (52.0%)   - Uruguay: 22/65 (34.0%)  - Italy: 11/19 (61.0%)  - Germany: 9/16 (56.0%)  - Netherlands: 13/16 (81.0%) -Salvesen et al. 1996: case study, 5/13 (38.5%) -Schmitt et al. 1981: case study, 1/1 (100.0%)  -Schober et al. 2001: case study, 2/2 (100.0%) -Shan et al. 2014: 14/34 (41.1%)  -Teh et al. 1997: case study, 1/1 (100.0%) -Tremolizzo et al. 2007: case study, no weakness reported -Vita et al. 1983: case study, 6/12 (50.0%) -Youssof, 2016: 75/89 (84.3%)  Atrophy of proximal lower limbs (hip, thigh), more frequently in case studies. Small sample size studies. Severity of atrophy ranging from mild to severe (Bosch et al. 1979, Fukuhara et al. 1982, Mihaylova et al. 2008, Muller et al. 2001, Nishii et al. 2021, Porschke et al. 1997, Salvesen et al. 1996, Schmitt et al. 1981, Teh et al. 1997, Vita et al. 1983).  Absence of weakness and/or atrophy reported in 3 studies (Piccolo et al. 2011, Ruegg et al. 2005, Tremolizzo et al. 2007)  No description regarding assessment method and standardization, choice of muscle groups assessed. Case-control studies in the majority. |
| **Patient file review** (n = 7) | **ClinRO:** Patient file review (n = 7) | Presence of proximal lower limb muscle weakness | Proximal lower limb weakness presents in 32/49 (65.3%) to **287/331 (86.7%)** patients. Retrospective studies with small to large sample sizes. No discussion on COA used to report weakness (Agarwal et al. 2012, Alonso-Pérez et al. 2022, Becher et al. 2001, Brisson et al. 2020, Jones et al. 2010, Youssof et al. 2015).  Proximal weakness (no precision) in 23/117 (19.7%) (Blumen et al. 1997). |
| **PerfO:** 21 different studies | | | |
| **Manual Muscle Testing (MMT)**  (n = 19) | **PerfO**: Medical Research Council Scale (MRC Scale) (n = 19) | Score of 0 to 5 for each muscle group assessed, where 0 indicates complete paresis and 5 normal muscle strength (n = 12) | Proximal lower limb weakness (hip flexors > hip extensors, abductors and adductors; knee extensors and knee flexors).  Weakness severity ranged from severe (MRC 2) to mild (MRC 4-4+), and weakness was reported in 5/16 (35.7%) to **34/40 (85.0%) participants**.  Precision of muscle groups assessed in a few studies (using only the term “proximal weakness”). Mostly case studies.  **Author’s last name, year: prevalence (%) and range of MMT score:**  -Blumen et al. 2013: 1/5 participant with MRC assessment (MRC 3 – MRC 5)  -Bosch et al. 1979: moderate weakness (MRC 4)  -Goyal et al. 2019: 13/25 (52.0%) with proximal limb weakness (MRC 2 – MRC 4)  -Mihaylova et al. 2008: 1/6 participant with proximal weakness and wasting (MRC 3)  -Nishii et al. 2021: deltoid (MRC 4), pectoralis major (MRC 3) and latissimus dorsi (MRC 4)  -Piccolo et al. 2011: no weakness reported (MRC 5)  -Saini et al. 2018: 1/4 participant with limb-girdle weakness (MRC 4-4+)  -Uyama et al. 1996: 3/10 participants with all proximal limb-girdle weakness (MRC 4- - 4+)  -Uyama et al. 1997: 5/6 participants with limb-girdle weakness (MRC 4- - 4+)  **-Van der Sluijs et al. 2016b: 34/40 (85.0%).** The weakness of the pelvic muscles varied from mild (MRC 4) to severe (MRC 2)  -Van der Sluijs et al. 2017: 5/16 (35.7%). Participant presenting with serratus anterior, deltoideus and triceps weakness (MRC score not specified). The muscle strength of the quadriceps muscles was preserved in 13/14 patients. Hip flexor weakness was present in 5/14 OPMD patients  -Vita et al. 1983: leg muscles were wasted and weak. Quadriceps, gastrocnemius and hamstring muscles were weak (grade 3-4 by Medical Research Council criteria). |
|  |  | Sum of score for each muscle or muscle group assessed (n = 3) | **Significant deterioration for total score of 14 muscles groups assessed, with four of them assessing proximal lower limbs (hip flexors and abductors, knee extensors and flexors)** (Kroon et al. 2021, Kroon et al. 2022).  Significant difference between OPMD and control groups (p < 0.05) for total sum score of 7 muscle groups, with three of them assessing proximal lower limbs (hip flexors, hip abductors and hip adductors) (Melkus et al. 2023).  No precision regarding MRC score of isolated muscle groups. Prospective studies with moderate sample size (25 to 43 patients). No assessment of hip extensors in the 3 studies. |
|  |  | Binomial: MRC ≤ 3 et MRC ≥ 4 (n = 1) | MRC ≤ 3 for hip flexors strength is associated to an earlier age of onset to use walking aid when compared to MRC ≥4 after control of age of disease onset and comorbidities (p<0.001). Hip flexors weakness is always present if another muscle group weakness is present (Youssof et al. 2015).  Use of hip flexors only in the analyses, which refrains conclusions for other muscle groups. |
|  |  | Number and % with weakness present (n = 1) | Proximal lower limb weakness presents in **33/108 (30.6%)** to 110/144 (76.4%), mostly symmetric (Alonso-Pérez et al. 2022). No precision regarding the muscle groups assessed with the MRC scale in the study, but available through request from the author. Retrospective study with large sample size (>100). |
|  |  | Mean and range for muscle group assessed (n = 1) | Proximal lower limb weakness presents in 13/16 (81.3%) of patients. Mean MRC score for hip (gluteus maximus, iliopsoas) and thigh (quadriceps only) muscles = MRC 3, range 1 – 5 (Van Der Sluijs et al. 2003). No results per muscle group, only the mean of these three muscle groups was presented. No assessment of hip abductors and adductors, and knee flexors. |
|  | **PerfO**: British Medical Research Council (MRC) (n = 1) | Score ranging from 0 to 5, where 0 means complete paresis and 5 normal muscle strength (n = 1) | Presence of atrophic paresis and predilection of distal muscle weakness at first, then progression to proximal lower limbs, legs and feet (MRC 4) (Schober et al. 2001). No precision regarding muscle groups impaired. No comparison with control group. Small sample size in a cohort design |
| **Quantitative Muscle Testing (QMT)** (n = 5) | **PerfO**: Fixed dynamometer KAP-S 2kN transducer; A.S.T. Angewandte System Technik GmbH, Dresden, Germany (n = 2) | Maximal isometric muscle contraction in kg. Best performance from three trials. | Significant deterioration of right quadriceps (knee extensors) (-12.7% (-3.7 to -21.7)), left quadriceps (-**14.3%** (-6.0 to -22.6)) and left iliopsoas (hip flexors) (-**12.2%** (-5.5 to -18.9)) over a period of 20 months (Kroon et al. 2021, Kroon et al. 2022). Only two muscle groups were assessed. Lever arm not taken into consideration. Results include both sexes and all age-span. Only the best performance is retained for analyses. Moderate sample size of 43 patients. No comparison to a control group or reference/normative values. |
|  | **PerfO:** Hand-held dynamometer (HDD) MedUp Atlas Medic (Quebec, Canada) (n = 1) | Maximal isometric muscle contraction in Newton-meter (Nm). Mean of closest 2 trials used for analyses. Comparison to predictive equations from Hogrel et al. 2007 taking into consideration age, sex and weight. | Significant difference between three age groups for proximal lower limb weakness (in Newton-meter; Nm) in four muscle groups (Brisson et al. 2023).  *Hip flexors (measures in Nm) (n=27)* Total (n = 27): 51.7 (29.4)  < 56 yo (n = 7): 66.2 (28.3) 56 – 65 yo (n = 8): 65.2 (24.7) > 65 yo (n = 12): 34.3 (24.8)  *Hip extensors (Nm) (n=26)* Total (n = 26): 150.4 (78.2)  < 56 yo (n = 7): 203.3 (82.0) 56 – 65 yo (n = 8): 166.0 (72.3) > 65 yo (n = 11): 105.3 (56.5)  *Knee flexors (Nm) (n=27)* Total (n = 27): 66.7 (29.9)  < 56 yo (n = 7): 86.7 (25.1) 56 – 65 yo (n = 8): 72.0 (25.6) > 65 yo (n = 12): 51.5 (28.8)  *Knee extensors (Nm) (n=26)*  Total (n = 26): 90.9 (38.6)  < 56 yo (n = 7): 115.8 (26.1) 56 – 65 yo (n = 8): 105.0 (36.5)  > 65 yo (n = 11): 64.7 (32.2)  Percentages from predictive equations of proximal lower limb weakness were different between three age groups and range between 65.9 to 98.3% (hip flexors and extensors, knee extensors and flexors)  *Hip flexors (% from reference values) (n=27)*  Total (n = 27); % from reference values: 65.9 (29.2)  < 56 yo: % from reference values: 83.0 (22.4)  56 – 65 yo: % from reference values: 78.2 (21.0)  > 65 yo: % from reference values: 46.0 (27.6)  *Hip extensors (% from reference values) (n=26)*  Total (n = 26); % from reference values: 98.3 (42.9)  < 56 yo: % from reference values: 147.9 (18.1)  56 – 65 yo: % from reference values: 100.8 (32.8)  > 65 yo: % from reference values: 71.6 (36.9)  *Knee flexors (% from reference values) (n=27):*  Total (n = 27); % from reference values: 114.5 (39.7)  < 56 yo: % from reference values: 144.1 (30.2)  55 – 65 yo: % from reference values: 117.8 (28.7)  > 65 yo : % from reference values: 93.1 (41.0)  *Knee extensors (% from reference values) (n=26)*  **Total (n = 26); % from reference values: 74.0 (23.7)**  < 56 yo: % from reference values: 88.6 (12.6)  56 – 65 yo: % from reference values: 80.9 (14.2)  > 65 yo: % from reference values: 59.7 (28.0) |
|  | **PerfO**: Non-specified strength gauge (n = 1) | Maximal voluntary contraction. Best of 3 attempts used in analyses. Identical lever-arm in every patient. | No significant difference between patients and control group for hip flexors, knee extensors and knee flexors (van der Sluijs et al. 2017). Results presenting one side only. Identical lever-arm between patients. Protocol presented. No comparison between reference/normative values. |
|  | **PerfO**: Portative dynamometer Citec CT 301 (n = 1) | Maximal isometric muscle contraction. Mean of three trials. Comparison to reference and normative values according to age and sex. | Proximal lower limb weakness more severe than upper proximal limb. No pattern retrieved concerning the predilection of flexors and extensors weakness. Individual variations were retrieved among patients. Approximation of mean percentage from normative values ranging from **50.0 to 57.5%**, according to age and sex of patients, for hip flexors **(50.0%)** and hip extensors, knee extensors and knee flexors **(55.0 to 57.5%)** (Witting et al. 2014). No assessment of hip abductors and hip adductors. Moderate sample size. No standardization of lever-arm. |

**S2 Table 2**: Key findings of COA’s reporting **distal lower limb muscle** weakness.

| **30 studies** | **COA** | **Method** | **Findings** |
| --- | --- | --- | --- |
| **PROM:** 2 different studies | | | |
| **Questionnaire**  (n = 2) | **PROM**: Home-made questionnaire (n = 2) | Standardized questionnaire before evaluation regarding OPMD symptoms | No weakness in distal lower limbs reported by patients (Van Der Sluijs et al. 2003, van der Sluijs et al. 2017). |
| **ClinRO:** 22 different studies | | | |
| **Clinical observation**  (n = 18) | **ClinRO**: Physical examination (n = 18) | Neurological examination of distal lower limbs | *Weakness in distal lower limbs* reported in 26/168 (15.5%) to **13/40 (32.5%)** of patients. Some studies precise muscle or muscle groups affected (Alonso-Jimenez et al. 2019, Creel et al. 1998, Fardeau et al. 1997, Fukuhara et al. 1982, Salvesen et al. 1996, Schmitt et al. 1981, Shan et al. 2014, van der Sluijs et al. 2016b, Vita et al. 1983).  *Weakness affecting the tibialis anterior, gastrocnemius and solear muscles* (Alonso-Jimenez et al. 2019, Blumen et al. 2013, Fardeau et al. 1997, Schober et al. 2001, Shan et al. 2014, van der Sluijs et al. 2016, Vita et al. 1983).  *Atrophy of upper or lower distal limbs* (Blumen et al. 2013, Mihaylova et al. 2008, Nishii et al. 2021, Salvesen et al. 1996, Schmitt et al. 1981, Vita et al. 1983). Absence of weakness in distal lower limb muscles (~~Bilgen et al. 2001~~, Blumen et al. 1993, Bosch et al. 1979, Nishii et al. 2021, Piccolo et al. 2011, Ruegg et al. 2005, Salvesen et al. 1996).  *Generalized weakness in limbs without precision*. Progression to distal limbs reported (Creel et al. 1998, Fardeau et al. 1997, Fukuhara et al. 1982).  **Author’s last name, year: prevalence (%):**  -Alonso-Jimenez et al. 2018: 26/168 (15.5%) -Bilgen et al. 2001: ¾ (75.0%) -Blumen et al., 1993: No cases of distal weakness among the 79 patients  -Blumen et al., 2013: distal weakness (tibialis anterior) reported with MRC Scale (see below) -Bosch et al. 1979: 0/1 (0%) -Creel et al. 1998: 1/2 (50.0%) (generalized limb weakness) **-**Fardeau et al. 1997: 15/29 (51.7%) (predominantly girdles, can expand to the lower limb extremities. Not taken into consideration for prevalence range since no precision if distal lower or upper limb are included in these -Fukuhara et al. 1982: 1/2 (50.0%) (muscle weakness more prominent in the peroneal muscles) -Mihaylova et al. 2008: 1/6 (16.7%) (marked atrophy of tibialis anterior muscle) -Nishii et al. 2021: 0% (no weakness in tibialis anterior and soleus muscles) -Piccolo et al. 2010: 0% -Ruegg et al. 2005: 0% (muscle strength was normal) -Salvesen et al. 1996: 0% -Shan et al. 2014: 9/34 (26.5%) (mild distal muscle involvement along with the disease’s progression but no precision if upper or lower limb, not taken into consideration for prevalence range). -Schmitt et al. 1981: 1/1 (100.0%). Lower limbs followed by upper limbs, with a distal predilection -Schober et al. 2001: 2/2 (100.0%) -**Van der Sluijs et al. 2016b: 13/40 (32.5%) (distal leg weakness)** -Vita et al. 1983: 2/2 (100.0%). Leg muscles atrophied and weak, particularly the anterior compartment. Mild bilateral foot drop. |
| **Patient file review** (n = 4) | **ClinRO**: Patient file review (n = 4) | Presence of distal muscle limb weakness in medical files | No distal limb weakness was reported in 14 patients (Jones et al. 2010). Assessed but not reported in a retrospective study (Brisson et al. 2020). Distal lower limb weakness in **2/52 (3.8%)** to 4/28 (14.3%) patients (Youssof et al. 2015).  *Alonsò-Pérèz et al. 2022:*  Canary cohort: There were no patients with distal limb weakness.  European GCN15: Distal muscle weakness was present in 50% of the patients.  European shorter cohort: Distal limb weakness was seen in European patients but not in patients from the Canary Islands (14 vs. 0 patients). |
| **PerfO:** 19 different studies | | | |
| **Manual Muscle Testing (MMT)** (n = 18) | **PerfO**: Medical Research Council Scale (MRC Scale) (n = 17) | Score ranges from 0 to 5, where 0 means complete paresis and 5 normal strength (n = 10) | Distal lower limb weakness (tibialis anterior, gastrocnemius and solear muscles) reported in three studies, ranging from moderate (MRC 3) to mild (MRC 4) and in combination with distal upper limb weakness (Mihaylova et al. 2008, van der Sluijs et al. 2016b). No weakness in distal lower limbs (MRC 5) reported in six studies (Bosch et al. 1979, Goyal et al. 2019, Nishii et al. 2021, Piccolo et al. 2011, Uyama et al. 1996, Uyama et al. 1997).  **Author’s last name, year: prevalence (%) and range of MRC score:**  -Blumen et al. 2013: 1/5 with tibialis anterior weakness and atrophy (MRC 4) -Bosch et al. 1979: strength in distal muscle preserved (MRC 5) -Goyal et al. 2019: no distal weakness reported using MRC in 25 participants (0.0%) (MRC 5)  -Mihaylova et al. 2008: 1/6 participants with tibialis anterior muscle weakness and atrophy (MRC 3)  -Nishii et al. 2021: no weakness in tibialis anterior or gastrocnemius (MRC 5) -Piccolo et al. 2011: no weakness  -Uyama et al. 1996: no distal lower limb muscle weakness in 10 participants, MRC 5  -Uyama et al. 1997: no distal lower limb muscle weakness in 6 participants, MRC 5 -Van der Sluijs et al. 2003: Assessed for tibialis anterior and soleus, weakness only reported from Figure 1 using a graph, no raw data available (approximately MRC 4 to MRC 5) -Van der sluijs et al. 2016b: **13/40 (32.5%)** distal leg weakness using MRC scale, no MRC score provided. One patient with MRC scale score: foot extensors and flexors (MRC 4). All others assessed also with MRC Scale but no MRC score nor severity reported  -Van der sluijs et al. 2017: 1/14 (7.3%) participants, distal leg weakness using MRC scale (no MRC score provided) -Vita et al. 1983: 2/2 (100.0%). MRC 1 of tibialis anterior and peronei muscles |
|  |  | Sum of muscle score, total score ranging from 0 to 130 (n = 2) | **Significant deterioration of total MRC scale score for 14 muscle groups over a period of 20 months. Two muscle groups assessed, distal lower limb weakness (ankle plantar flexors and dorsiflexors)** (Kroon et al. 2021, Kroon et al. 2022) Results only presented for total sum of muscle score, no isolated muscle groups discussed. Moderate sample size. No comparison to control groups nor normative/reference values. |
|  |  | Number and % of patients with weakness (n = 5) | Distal muscle limb (tibialis anterior, gastrocnemius and solear) weakness ranging from moderate (MRC 3) to mild (MRC 4) in **2/52 (3.8%)** to 4/28 (14.3%) patients (Alonso-Pérez et al. 2022, Van Der Sluijs et al. 2003, Vita et al. 1983, Youssof et al. 2015). No prevalence reported in retrospective study (Brisson et al. 2020). No raw data reported in a cross-sectional study (Van Der Sluijs et al. 2003). Retrospective and case studies. |
|  | **PerfO**: British Medical Research Council (MRC) (n = 1) | Score ranging from 0 to 5, where 0 means complete paresis and 5 normal strength | Mild muscle weakness (MRC 4) in leg muscles with a distal predilection in a case study (Schober et al. 2001). No precision on muscle groups affected. |
| **Quantitative Muscle Testing (QMT)**  (n = 1) | **PerfO**: Portative dynamometer Citec CT 301 (n = 1) | Maximal isometric muscle contraction. Mean of three trials. Comparison to age and sex-matched controls. | Distal lower limb (ankle plantar flexors and ankle dorsiflexors) weakness **around 60.0%** **of the reference values.** Similar weakness between plantar flexors and dorsiflexors (Witting et al. 2014). Distal lower limb more severe than distal upper limb. No predilection of flexors and extensors weakness. Weakness characterized by individual variations. |

**S2 Table 3**: Key findings of COA’s reporting **proximal upper limb muscle** weakness.

| **50 studies** | **COA** | **Method** | **Findings** |
| --- | --- | --- | --- |
| **PROM:** 5 different studies | | | |
| **Interview**  (n = 1) | **PROM:** Semi-structured interview (n = 1) | « Framework » technic | Proximal upper limb weakness reported in 12/25 (48.0%) participants (Kurtz et al. 2019). Weakness affects activities of daily living. No precision on muscle groups affected. |
| **Questionnaire**  (n = 4) | **PROM**: Homemade socio-demographic questionnaire (n = 4) | Standardized questionnaire before the evaluation regarding OPMD symptoms. | Proximal upper limb weakness reported in **9/35 (25.7%)** to **13/16 (81.3%) participants.** Inability to raise arms above the head or to comb hair (Tremblay-Tymczuk et al. 1992, Van Der Sluijs et al. 2003, van der Sluijs et al. 2017, van der Sluijs et al. 2016a). No precision regarding muscle groups considered as “weak”. Small to moderate sample sizes. |
| **ClinRO:** 36 different studies | | | |
| **Clinical observation**  (n = 29) | **ClinRO**: Clinical observation (n = 29) | Neurological examination of proximal upper limb muscle strength (n = 29) | Proximal upper limb weakness (deltoids, brachial biceps and triceps, and periscapular muscles) with or without proximal lower limb weakness ranging from 2/49 (4.1%) to 53/89 (59.6%) of patients. Symetrical > asymetrical weakness and varying from mild to severe.  **Author’s last name, year: prevalence (%):**  -Alonso-Jimenez: 48/168 (28.6%) -Bilgen et al. 2001: case study, 3/4 (75.0%) (proximal weakness, predominant in proximal thigh) -Blumen 2013: case study, 5/5 (100.0%) -Blumen 1993: Lower OR upper and lower proximal weakness: 21/79 (27.0%) -Bosch et al. 1979: case study, 1/1 (100.0%) -Creel et al 1998: case study, 1/2 (50.0%). Generalized limb weakness -Fardeau et al. 1997: 15/29 (51.7%) (predominantly girdles, can spread to lower limbs) -Fukuhara et al. 1982: case study, 2/2 (100.0%) -Goyal et al., 2019: 13/25 (52.0%) (proximal weakness, no precision) -Hill et al. 2001: 3/31 (9.7%) -Kuo et al., 2009: case study, 1/10 (10.0%) -Medici et al., 1997: Proximal weakness, mainly lower limbs 22/65 (33.9%) -Mihaylova et al., 2008: case study, 1/6 (16.7%) -Muller et al. 2001: 9/16 (56.3%) -Munitiz et al., 2003: case study, 1/3 (33.3%) -Nagashima et al., 2000: case study, 3/4 (75.0%) (no precision, proximal weakness) -Nishii et al., 2021: case study, 1/1 (100.0%) -Piccolo et al., 2011: case study, 0/1 (0.0%) -Porschke et al., 1997: No prevalence reported, but assessed. Presence of progressive pareses and wasting of limb girdle muscles -Robinson et al. 2011: case study, 0/2 (0.0%) -Ruegg et al., 2005: case study, 0/1 (0.0%) -Salvesen et al., 1996: case study, 5/13 (38.5%) -Schmitt et al., 1981: case study, 1/1 (100.0%) -Schober et al. 2001: case study, 1/1 (100.0%) -Shan et al. 2014: 14/34 (41.2%) (proximal weakness, no precision) -Teh et al. 1997: case study, 1/1 (100.0%) (proximal weakness, no precision) -Tremolizzo et al., 2007: case study, 1/1 (100.0%) -Vita et al., 1983: case study, 1/12 (8.3%) -Youssof, 2016: 53/89 (59.6%)  Atrophy or dystrophy of muscles from shoulders and elbow. Symetrical > asymetrical scapular winging and scapular girdle weakness. Case studies > cross-sectional > retrospective studies. Sample sizes range from small to large (Blumen et al. 2013, Bosch et al. 1979, Fukuhara et al. 1982, Goyal et al. 2019, Mihaylova et al. 2008, Muller et al. 2001, Munitiz et al. 2003, Nishii et al. 2021, Piccolo et al. 2011, Porschke et al. 1997, Salvesen et al. 1996, Schmitt et al. 1981, Teh et al. 1997, Vita et al. 1983).  No proximal upper limb weakness in three studies (case studies and review) (Piccolo et al., 2011, Robinson et al. 2011, Ruegg et al. 2005). |
| **Patient file review** (n = 7) | **ClinRO**: Patient file review (n = 7) | Incapacity to elevate arms over shoulders (n = 1) | Incapacity to elevate arms over the shoulders reported in **11/303 (3.6%) participants.** Retrospective design (Brisson et al. 2020). |
|  |  | Presence of proximal upper limb weakness (n = 6) | Proximal limb weakness reported from medical files without precision if the weakness is for lower or upper limb, ranging from 3/49 (6,1%) to **12/14 (85.7%)** (Agarwal et al. 2012, Alonso-Pérez et al. 2022, Becher et al. 2001, Blumen et al. 1997, Jones et al. 2010, Youssof et al. 2015). No assessment of weakness severity.  *Alonsò-Pérèz et al. 2022:* Canary cohort: Proximal upper limb weakness was present in 16 patients (14.8%) at a mean age of 60.4 years (range 47–69).  European GCN15: 37.5%: proximal weakness upper limbs  European short GCN: Proximal upper limb weakness was present in 42 (35.3%) patients that was also more frequent than in the Canary (GCN)15 cohort (p= 0.001, Chi-squared test). |
| **PerfO:** 22 different studies | | | |
| **Manual Muscle Testing (MMT)** (n = 19) | **PerfO**: Medical Research Council Scale (MRC Scale) (n = 18) | Score ranges from 0 to 5, where 0 means complete paresis and 5 normal strength (n = 9) | Proximal upper limb weakness ranging from 1/14 (7.1%) to **22/40 (55.0%)** of patients. Few studies specify the affected muscles: deltoids, pectoralis major, latissimus dorsi, supraspinatus, infraspinatus, brachial biceps and triceps (Bosch et al. 1979, Nishii et al. 2021, van der Sluijs et al. 2017). Weakness, when present, ranges from mild (MRC 4-4+) to moderate (MRC 2) (Blumen et al. 2013, Bosch et al. 1979, Goyal et al. 2019, Mihaylova et al. 2008, Nishii et al. 2021, Saini et al. 2018, van der Sluijs et al. 2017) and is present with proximal lower limb weakness in some cases (Mihaylova et al. 2008, Uyama et al. 1996, Uyama et al. 1997). Case studies > cross-sectional > retrospective studies. Small to large sample sizes.  **Author’s last name, year: prevalence (%) and range of MRC scale score:**  -Blumen et al. 2013: case study, 1/5 participant with MRC assessment (MRC 3 – MRC 5)  -Bosch et al. 1979: case study, moderate weakness (MRC 4)  -Goyal et al. 2019: 13/25 (52.0%) with proximal limb weakness (MRC 2 – MRC 4)  -Mihaylova et al. 2008: 1/6 participant with proximal weakness and wasting (MRC 3)  -Nishii et al. 2021: case study, deltoid (MRC 4), pectoralis major (MRC 3) and latissimus dorsi (MRC 4)  -Saini et al. 2018: case study, 1/4 participant with limb-girdle weakness (MRC 4-4+)  -Uyama et al. 1996: case study, 5/10 participants with limb-girdle weakness (MRC 4- - 4+)  -Uyama et al. 1997: case study, 5/6 participants with limb-girdle weakness (MRC 4- - 4+)  -Van der Sluijs et al. 2017: Assessed using MMT, but no prevalence reported and no MRC score specified |
|  |  | Sum of score for each muscle group (composite score) (n = 3) | **Significant decline of the total MRC score for 14 muscle groups over a period of 20 months (−2.5% (−0.9 to −4.1)), which includes three muscle groups for proximal upper limb weakness: shoulder abductors, elbow flexors and elbow extensors (Kroon et al. 2021, Kroon et al. 2022).**  Significant difference between OPMD patients and controls for 7 muscle groups (which include four proximal upper limb muscles: shoulder abductors, shoulder external rotators, elbow flexors and elbow extensors) (Melkus et al. 2023).  No raw data per muscle groups presented. |
|  |  | Number and % with weakness present (n = 4) | Proximal upper limb weakness (shoulder abductors, elbow flexors and elbow extensors) reported in **2/37 (5.4%)** to 44/130 (33.8%) of patients and ranging from mild (MRC 4) to severe (MRC 2). Absence of weakness (MRC 5) is also reported (Alonso-Pérez et al. 2022, van der Sluijs et al. 2016b, Youssof et al. 2015). Not presented but assessed in one study (Brisson et al. 2020) |
|  |  | Mean and range for MRC scores (n = 2) | Proximal upper limb weakness (deltoids, pectoralis major, brachial biceps and triceps) was present and ranging from mild (MRC 4) to moderate (MRC 3) (Van Der Sluijs et al. 2003, Vita et al. 1983). No score for isolated muscles reported. |
|  | **ClinRO**: British Medical Research Council (MRC Scale) (n = 1) | Score ranges from 0 to 5, where 0 means complete paresis and 5, normal strength | Mild proximal upper limb weakness with muscle atrophy and distal predilection (Schober et al. 2001). No precision regarding muscle groups impaired. |
| **Quantitative Muscle Testing (QMT)** (n = 4) | **PerfO:** Fixed dynamometry KAP-S 2kN transducer; A.S.T. Angewandte System Technik GmbH, Dresden, Germany (n = 2) | Maximal isometric muscle contraction. Best attempts from 3 trials used for analyses. | Significant deterioration of deltoid muscle strength ranging from –**23.5% (-16.8 to –30.2**) **for the right side to** **–26.8% (-16.7 to –36.9) for the left side** (Kroon et al. 2021, Kroon et al. 2022). No lever-arm standardization. No separation between patients’ sex and age. |
|  | **PerfO:** Hand-held dynamometer (HHD) MedUp Atlas Medic (Quebec, Canada) (n = 1) | Maximal isometric muscle contraction. Mean of closest two attemps. Measures in Netwon-meter (Nm). | **Significant difference only for shoulder flexors between three age groups**. No comparison to reference/normative values (Brisson et al. 2023).  *Shoulder flexors (Nm), n=26:*  Total (n = 26): 49.3 (27.8)  < 56 yo (n = 7): 64.5 (30.0)  56 – 65 yo (n = 8): 60.2 (23.4)  > 65 yo (n = 11): 31.8 (20.3)  *Shoulder abductors (Nm), n=26:*  Total (n = 26): 45.1 (19.3)  < 55 yo (n = 7): 56.6 (24.4)  55 – 65 yo (n = 7): 44.9 (11.5)  > 65 yo (n = 12): 38.5 (18.0)  *Elbow flexors (Nm), n=27:*  Total (n = 27): 37.0 (18.0)  < 55 yo (n = 7): 46.2 (16.0)  55 – 65 yo (n = 8): 41.5 (14.7) > 65 yo (n = 12): 28.6 (18.6) |
|  | **PerfO**: Dynamomètre portatif Citec CT 301 (n = 1) | Maximal isometric muscle contraction. Mean of three trials. Comparison to age and sex-matched controls. | **Mild (less than 1 standard deviation) to severe (more than 2 standard deviation) proximal upper limb weakness (elbow flexors > elbow extensors) characterized by individual variations. Approximative percentage from reference values with age and sex-matched controls ranged between 61.0 and 96.0% for** shoulder abductors, elbow flexors and elbow extensors (Witting et al. 2014). No lever-arm standardization. No specific result for isolated muscle group discussed in the “results” section (only a histogram present). |
| **Other test**  (n = 1) | **PerfO:** homemade standardized test | Capacity to raise the right arm, left arm and both arms over the shoulder for 10 seconds | Eighteen people could lift and hold their left arm in the air without difficulty, three could with difficulty, one could but found it very difficult (difficulty in **4/22 patients (18.2%)**. Eighteen people could raise and hold the right arm and then the two arms together without difficulty, two with a little difficulty, one with a lot of difficulty, and one was not able to (Tremblay-Tymczuk et al. 1992). |

**S2 Table 4**: Key findings of COA’s reporting **distal upper limb** muscle weakness.

| **34 studies** | **COA** | **Method** | **Findings** |
| --- | --- | --- | --- |
| **PROM:** 3 different studies | | | |
| **Interview**  (n = 1) | **PROM:** Semi-structured interview (n = 1) | « Framework » technic | Hand weakness in **1/25 (4.0%)** participants and impaired dexterity in **1/25 (4.0%)** participants. Difficulty in lacing shoes, cooking, opening container and handles (Kurtz et al. 2019). No precision regarding muscles or muscle groups impaired. |
| **Questionnaire**  (n = 2) | **PROM**: Homemade socio-demographic questionnaires (n = 2) | Questionnaire  before the evaluation to assess OPMD symptoms. | No muscle weakness reported for distal upper limbs among participants (Van Der Sluijs et al. 2003, van der Sluijs et al. 2017). |
| **ClinRO:** 23 different studies | | | |
| **Clinical observation**  (n = 19) | **ClinRO**: Physical examination of distal upper limb strength (n = 19) | Neurological examination of muscle strength in the distal upper limb | Distal upper limb weakness was reported in 14/168 (8.3%) to **10/40 (25.0%)** patients. Some studies specify muscles or muscle groups impaired (Alonso-Jimenez et al. 2019, Bilgen et al. 2001, Blumen et al. 2013, Blumen et al. 1993, Creel et al. 1998, Fardeau et al. 1997, Fukuhara et al. 1982, Mihaylova et al. 2008, Nishii et al. 2021, Piccolo et al. 2011, Ruegg et al. 2005, Salvesen et al. 1996, Schmitt et al. 1981, Schober et al. 2001, Shan et al. 2014, Uyama et al. 1996, Uyama et al. 1997, van der Sluijs et al. 2016b, Vita et al. 1983).  **Author’s last name, year: prevalence (%):** -Alonso-Jimenez et al.: 14/168 (8.3%) -Blumen et al. 2013: case study, 1/5 (20.0%). Distal ulnar weakness and moderate atrophy of first interossei -Blumen et al. 1993: No cases of distal weakness among the 79 patients -Bilgen et al. 2001: case study, 0/1 (0%) -Bosch et al. 1979: case study, 0/1 (0%) -Creel et al. 1998: case study, 50.0% (limb weakness not specified) -Fardeau et al. 1997: 15/29 (51.7%) (lack of precision, only mentionned a progression from limb-girdles to lower limb extremities: not taken into consideration for prevalence range) -Fukuhara et al. 1982: case study, 1/2 (50.0%) -Mihaylova et al. 2008: case study, 1/6 (16.7%) -Nishii et al. 2021: case study, 0/1 (0%) -Piccolo et al. 2011: case study, 0/1 (0%) -Ruegg et al. 2005: case study, 0/1 (0%) -Salvesen et al. 1996: case study, 0/13 (0%) -Schober et al. 2001: case study, 2/2 (100.0%) -Schmitt et al. 1981: case study, 1/1 (100.0%), weakness and wasting occurred predominantly in his lower limbs, and subsequently his arms became involved less severely in a pronounced distal distribution. -Shan et al. 2014: 9/34 (26.5%) (distal, but no precision if upper or lower limb, not taken into consideration for prevalence range) -Uyama et al. 1996: case study, 0/10 (0%) -Uyama et al. 1997: case study, 0/6 (0%) -Vita et al. 1983: case study, 0/2 (0%), no weakness of distal upper limb muscles  Weakness in 1st dorsal interosseous, muscles from thenar eminence, wrist flexors and wrist extensors) (Alonso-Jimenez et al. 2019, Blumen et al. 2013, Fardeau et al. 1997, Schober et al. 2001, Shan et al. 2014, van der Sluijs et al. 2016b)  Atrophy of distal upper limbs (Blumen et al. 2013, Mihaylova et al. 2008, Nishii et al. 2021, Salvesen et al. 1996, Schmitt et al. 1981, Schober et al. 2001, Vita et al. 1983).  Generalized limb weakness without precision of muscles groups impaired. Devlopment to distal limb weakness reported (Creel et al. 1998, Fardeau et al. 1997, Fukuhara et al. 1982, Shan et al. 2014). |
| **Patient file review** (n = 4) | **ClinRO**: Patient file review (n = 4) | Presence of distal upper limb weakness | Distal upper limb weakness reported in **2/38 (5.3%)** to 4/28 (14.3%) of the patients (Youssof et al. 2015). No distal upper limb weakness in 14 patients (Jones et al. 2010).  No precision if distal upper or lower limb in one study (Alonso-Pérez et al. 2022).  Assessed, but not reported a large retrospective study (Brisson et al. 2020). |
| **PerfO:** 20 different studies | | | |
| **Manual Muscle Testing (MMT)** (n = 17) | **PerfO**: Medical Research Council Scale (MRC Scale) (n = 16) | Score ranges from 0 to 5, where 0 means complete paresis and 5 normal strength (n = 10) | Distal upper limb weakness (thenar eminence, wrist flexors/extensors) presents with severity ranging from mild (MRC 4) to moderate (MRC 3) in **up to 25.0%** of patients (Blumen et al. 2013, Goyal et al. 2019, Mihaylova et al. 2008, Van Der Sluijs et al. 2003, van der Sluijs et al. 2016b, Vita et al. 1983).  **Author’s last name, year: prevalence (%) and range of MMT score:** -Blumen et al. 2013: Case study, 2/5 distal ulnar muscle weakness (MRC 4) -Bosch et al. 1979: Case study, no weakness (MRC 5) reported in distal muscle groups using MRC scale -Goyal et al. 2019: No weakness (MRC 5) in distal upper limb muscles reported in 25 participants using MRC scale -Mihaylova et al. 2008: Case study, 1/6 atrophy and weakness of thenar muscles (MRC 3) -Nishii et al. 2021: Case study, no weakness reported in distal upper limb muscles (MRC 5) -Uyama et al. 1996: Case study, not assessed in four participants, no weakness in distal upper limbs reported in remaining 6 participants (MRC 5) -Uyama et al. 1997: Case study, no weakness in distal upper limbs reported in remaining 6 participants (MRC 5) -Van der Sluijs et al. 2003: Assessed for extensors and flexors of the hand, weakness only reported from Figure 1 using a graph, no raw data available (approximately MRC 4 to MRC 5) -Van der Sluijs et al. 2016b: **10/40 (25.0%)** participants with distal arm weakness, assessed with MRC Scale but no MRC score nor severity reported -Vita et al. 1983: Case study, MRC scale, no weakness in upper distal limbs  No distal upper limb weakness reported (MRC 5) (Bosch et al. 1979, Brisson et al. 2020, Goyal et al. 2019, Nishii et al. 2021, Uyama et al. 1996, Uyama et al. 1997, Vita et al. 1983). |
|  |  | Sum of score for each muscle or muscle group (composite score) (0 - 130) (n = 2) | Significant deterioration over a period of 20 months for the summation of MRC Scale for 14 muscle groups. Three distal upper limb muscle groups (grip strength, wrist flexors and wrist extensors) (Kroon et al. 2021, Kroon et al. 2022). No precision regarding isolated muscle group. Moderate sample size. |
|  |  | Number and % with distal upper limb weakness (n = 5) | Distal upper limb weakness (wrist flexors and wrist extensors) with a severity ranging from mild (MRC 4) to moderate (MRC 3) present in **2/38 (5.3%)** to 4/28 (14.3%) patients (Alonso-Pérez et al. 2022, Van Der Sluijs et al. 2003, Youssof et al. 2015). No raw data reported in a cross-sectional study (van der Sluijs et al. 2017). Assessed but not reported in a retrospective study (Brisson et al. 2020). Retrospective study and case studies. |
|  | **PerfO**: British Medical Research Council Scale (MRC Scale) (n = 1) | Score ranges between 0 to 5, where 0 means complete paresis and 5 means normal strength (n = 1) | No weakness reported in distal upper limb weakness (Schober et al. 2001). No precision regarding muscle groups. |
| **Quantitative Muscle Testing (QMT)**  (n = 3) | **PerfO**: Unspecified grip dynamometer (n = 1) | Mean of 3 trials per hands | Results not reported due to an absence of distal upper limb weakness in OPMD. Study from 1992 (Tremblay-Tymczuk et al. 1992). |
|  | **PerfO:** Hand-Held Dynamometer (HHD). Jamar (Performance Health, Mississauga, Canada) (n = 1) | Maximal grip strength. Mean of 3 values. | *Grip strength (Kg), n=29.* Significant difference between 3 age groups (Brisson et al. 2023) Total (n = 29): 26.2 (11.4)  < 55 yo (n = 9): 34.3 (11.5)  55 – 65 yo (n = 8): 25.6 (8.9)  > 65 yo (n = 12): 20.5 (9.7)  *Grip strength (% from reference values), n=29.* **Significant difference between three age groups.**  Total (n = 29), **67.1 (21.2)**  < 55 yo: 82.3 (20.0)  55 – 65 yo: 63.9 (17.9) > 65 yo: 57.8 (18.9) |
|  | **Perfo:** Baseline pinch gauge. Jamar (Performance Health, Mississauga, Canada) (n = 1, (same study)) | Maximal pinch strength. Mean of 3 values. | *Pinch strength (Kg), n=30.* Significant difference between 3 age groups (Brisson et al. 2023). Total (n = 30): 5.8 (2.1)  < 55 yo (n = 9): 7.2 (1.9)  55 – 65 yo (n = 9): 6.0 (2.1)  > 65 yo (n = 12): 4.6 (1.6)  *Pinch strength (% from reference values), n=30.* **Significant difference between three age groups.**  Total (n = 30); **73.3 (20.8)**  < 55 yo: 88.3 (17.2)  55 – 65 yo: 69.9 (20.1)  > 65 yo: 64.7 (18.8) |
|  | **PerfO**: Grip dynamometer Smedley, Stoeltinger (n = 1) | Mean of three measures. Comparison with age and sex-matched controls. | Grip strength approximately 71% of the reference value, with **7/13 participants (53.8%)** with a **reduced grip strength of one or more standard deviations from the control group** (Witting et al. 2014). No raw data presented in the study, only available through supplementary table. |

**S2 Table 5**: Key findings of COA’s reporting **axial muscle** weakness.

| **32 studies Ptosis: 50 studies** | **COA** | **Method** | **Findings** |
| --- | --- | --- | --- |
| **PROM**: 5 different studies | | | |
| **Interview**  (n = 1)  **Ptosis** (n = 1) | **PROM**: Semi-structured interview (n = 1) | « Framework » technic | Axial limb weakness reported for **cervical/neck muscles 5/25 (20.0%), facial 2/25 (8.0%) and ocular 19/25 (76.0%) muscles** (Kurtz et al. 2019). Ptosis self-reported in 24/25 (96.0%). |
| **Questionnaire**  (n = 4) **Ptosis** (n = 4) | **PROM**: Homemade socio-demographic questionnaire (n = 4) | Questionnaire before the evaluation regarding OPMD symptoms | Ptosis frequently reported as spontaneously reported complaints **in up to 17/35 (49.0%)** patients. No complaints regarding other axial muscles (Van Der Sluijs et al. 2003, van der Sluijs et al. 2016a, van der Sluijs et al. 2017, van der Sluijs et al. 2016b). |
| **ClinRO:** 17 different studies  47 different studies using a **ClinRO** to assess ptosis or external eye muscle weakness | | | |
| **Clinical observation**  (n = 16) **Ptosis or external eye muscle weakness** (n = 40) | **ClinRO**: Physical examination of axial muscle strength (n = 16) Including ptosis and external eye muscles (n = 40) | Neurological examination of muscle strength for axial muscle strength (neck, facial, trunk and abdominal muscles)  - Facial, abdominal and trunk muscles (Total: n = 16, including 6 studies for prevalence and 10 case studies not taken into consideration for prevalence).  - ptosis, external eye muscles (n = 40) | *Neck flexors and/or extensors weakness*, symmetrical and reported in up to 21/168 (12.5%) of participants (Alonso-Jimenez et al. 2019, Goyal et al. 2019, Nagashima et al. 2000, Nishii et al. 2021, Robinson et al. 2011). Severe neck muscle rigidity reported.  **Author’s last name, year: prevalence (%):** -Alonsò-Jimenez et al. 2019: 21/168 (12.5%) -Shan et al. 2014: no weakness reported among 34 participants in neck flexors or extensors -Bosch et al. 1979: case study, neck thin with neck flexors and sternocleidomastoids weak  -Fukuhara et al. 1982: case study, the sternocleidomastoid was mildly weak on the right side  -Nagashima et al. 2000: case study, 3/4 participants presenting severe neck rigidity  -Ruegg et al. 2005: case study, astrologist posture to compensate from ptosis  -Vita et al. 1983: case study, no weakness reported in neck flexors or extensors reported    *Neck and/or facial muscles atrophy*  **Author’s last name, year: prevalence (%):** -Alonsò-Jimenez et al. 2019: 42/168 (25.0%) -Mihaylova et al. 2008: 1/6 (16.7%)  -Nishii et al. 2021: case study, neck weakness and rigidity  -Robinson et al. 2011: case study, 1/2 participants with severe neck flexion No weakness in 3 studies (Bilgen et al. 2001, Piccolo et al. 2011, Ruegg et al. 2005).  *Facial muscle weakness*, asymmetric > symmetric, varying from **2/16 (12.5%) to** **13/16 (81.3%)** patients. Mild to moderate weakness. **Author’s last name, year: prevalence (%):**  -Witting et al. 2014: **13/16 (81.3%)**  -Muller et al. 2001: **2/16 (12.5%)** facial weakness (not specified)  -Porschke et al. 1997: facial muscle weakness presence in some participants, no severity nor prevalence reported.  -Salvesen et al. 1996: case study, 4/13 (30.8%) with weak facial muscles  -Schmitt et al. 1981: case study, no weakness of neck muscles nor facial muscles  *Trunk muscle weakness/atrophy* **Author’s last name, year: prevalence (%)**: -Alonso-Jimenez et al. 2018: only reported from MRI, paraspinal > abdominal muscles -Porschke et al. 1997: presence of trunk muscle weakness (no prevalence reported) -Piccolo et al. 2011: case study, no weakness reported for trunk muscles  *Abdominal muscle weakness/atrophy* **Author’s last name, year: prevalence (%):** -Alonsò-Jiménez et al. 2018: not reported using COA, only reported from MRI (paraspinal > abdominal muscles) (Alonso-Jimenez et al. 2019)  *Weakness of levator palpebrae superioris (ptosis)* present from 37.5 to 100.0% of patients (Alonso-Jimenez et al. 2019, Alonso-Pérez et al. 2022, Blumen et al. 2013, Blumen et al. 1993, Bosch et al. 1979, Creel et al. 1998, Dobrowski et al. 1986, Eustace et al. 1989, Fardeau et al. 1997, Fischmann et al. 2011, Fukuhara et al. 1982, Goyal et al. 2019, Mihaylova et al. 2008, Muller et al. 2001, Porschke et al. 1997, Saini et al. 2018, Salvesen et al. 1996, Teh et al. 1997, Uyama et al. 1996, Uyama et al. 1997, Van Der Sluijs et al. 2003, van der Sluijs et al. 2016a, van der Sluijs et al. 2017, van der Sluijs et al. 2016b, Witting et al. 2014). **Author’s last name, year: prevalence (%):** -Alonso-Jimenez et al. 2018; 142/168 (84.5%) -Bilgen et al. 2001: case study, 3/4 (75.0%);  -Blumen et al. 1993; 77/79 (97.5%) -Blumen et al. 2013; case study, 5/5 (100.0%) -Bosch et al. 1979: case study, 1/1 (100.0%)  -Creel et al. 1998: case study, 2/2 (100.0%)  -Dobrowski et al. 1986; case study, 1/1 (100.0%) -Eustace et al. 1982; case study, 3/8 (37.5%) -Fardeau et al. 1997: 28/29 (96.6%)  -Fischmann et al. 2011; 7/8 (87.5%)  -Fukuhara et al. 1982: case study, ½ (50.0%);  -Goyal et al. 2019; 25/25 (100.0%)  -Kuo et al. 2009; 6/10 (60.0%) -Medici M. et al. 1997; 43/65 (66.2%)  -Mihaylova et al. 2008: 6/6 (100.0%) -Muller et al. 2001; 16/16 (100.0%);  -Munitiz et al. 2003; 3/3 (100.0%) -Nagashima et al. 2000: case study, 4/4 (100.0%);  -Nishii et al 2021: case study, 1/1 (100%) -Porschke et al. 1997; assessed, no prevalence reported -Robinson et al. 2011; case study, 2/2 (100.0%) -Ruegg et al. 2005; case study, 1/1 (100.0%) -Saini et al. 2018; case study, 4/4 (100.0%) -Salvesen et al. 1996; case study, 13/13 (100.0%) -Schmitt et al. 1981; case study, 1/1 (100.0%) -Schober et al. 2001; case study, 2/2 (100.0%) -Shan et al. 2014; case study, 27/34 (79.4%) -Teh et al. 1997: case study, 1/1 (100.0%) -Tremblay-Tymczuk, 1992: 21/22 (95.5%) -Tremolizzo et al. 2007: 1/1 (100.0%) -Uyama et al. 1996; case study, 10/10 (100.0%) -Uyama et al. 1997; case study, 6/6 (100.0%) -van der Sluijs et al. 2003: 16/16 (100.0%) -van der Sluijs et al. 2016a; 17/35 (49.0%) -van der Sluijs et al. 2016b; 35/40 (88.0%)  -van der Sluijs et al. 2017; 7/14 (50.0%) -Witting et al. 2014; 13/13 (100.0%)  -Youssof 2016: 83/89 (93.3%)  *External eye muscle weakness/atrophy* **Author’s last name, year: prevalence (%):** -Blumen et al. 1993; 23/79 (29.0%) -Blumen et al. 2013; case study, 2/5 (40.0%) (3/5 not assessed in study) -Fardeau et al. 1997; 16/29 (55.2%) -Fukuhara et al. 1982; case study, 0/2 (0%) -Porschke et al. 1997; In a family of 25 patients, prevalence NR, but presence of weakness for external eye muscle -Piccolo et al. 2011: 1/1 (100.0%) -Muller et al. 2001: 2/16 (12,5%);  -Robinson et al. 2011; case study, 2/2 (100.0%) -Saini et al. 2018; case study, 1/4 (25.0%) -Salvesen et al. 1996: case study, 7/8 (87.5%) -Teh et al. 1997: case study, 0/1 (0%) -Uyama et al. 1996; case study, 0/10 (0%) -Uyama et al. 1997; case study, 0/6 (0%) |
| **Patient file review** (n = 1)  **Ptosis (n = 7)** | Patient file review (n = 7) | Presence of ptosis, external ophtalmoplegia, facial, trunk or abdominal weakness | *Facial weakness*  Presence of facial weakness in 20/117 (17.1%) of participants (Blumen et al. 1997).  *Ptosis*  Presence of ptosis reported in most patients, up to 115/117 (98.0%) (Agarwal et al. 2012, Alonso-Pérez et al. 2022, Becher et al. 2001, Blumen et al. 1997, Brisson et al. 2020, Jones et al. 2010, Youssof et al. 2015).  No abdominal, trunk or neck muscle weakness reported using patient file review. |
| **PerfO:** 15 different studies | | | |
| **Manual Muscle Testing (MMT)** (n = 14) | **PerfO**: Medical Research Council Scale (MRC Scale) (n = 14) | Score ranges from 0 to 5, where 0 means complete paresis and 5 normal strength (n = 11) | *Axial muscle weakness (sternocleidomastoids, neck flexors and neck extensors)* reported, ranging **between severe (MRC 2) and MRC 5 (neck flexors > neck extensors) from 1/25 (4.0%) to 19/40 (47.5%) of participants** (Alonso-Pérez et al. 2022, Blumen et al. 2013, Goyal et al. 2019, Nishii et al. 2021, Saini et al. 2018, Uyama et al. 1996, Uyama et al. 1997, Van Der Sluijs et al. 2003, van der Sluijs et al. 2017, van der Sluijs et al. 2016b, Witting et al. 2014). No precision on muscle or muscle groups reported in some studies.  **Author’s last name, year: prevalence (%):**  -Alonsò-Perez et al. 2022: MRC scale assessed, but no prevalence reported for axial weakness -Blumen et al. 2013: case study, 1/5 participant weakness in sternocleidomastoids (MRC 4)  **-Goyal et al. 2019: 1/25 (4.0%)** participant with neck weakness (MRC 3)  -Nishii et al. 2021: case study, weakness in neck extensors (MRC 4) and neck flexors (MRC 2) -Uyama et al. 1996: case study, MRC not assessed in 4/10 participants. 5/6 participants with neck weakness (MRC 4- to 4+) -Uyama et al. 1997: case study, 5/6 participants with neck weakness (MRC 4- to 4+)  **-Van der Sluijs et al. 2016b:** Neck flexor weakness and neck extensor weakness occurred in respectively **19 (47.5%)** and in 11 (27.5%) patients. No MRC severity reported. -Van der Sluijs et al. 2017: MRC scale of neck flexors and neck extensors assessed, but no severity nor prevalence reported.  *Facial muscle weakness* present in **3/25 (12.0%)** **to 11/13 (84.6%)** patients and ranging from moderate (MRC 3) to mild (MRC 4) (Goyal et al. 2019, Saini et al. 2018, van der Sluijs et al. 2016b, Witting et al. 2014).  -Goyal et al. 2019: **3/25 (12.0%)** participants with facial weakness (MRC 3)  -Saini et al. 2018: case study, 1/4 participants with mild facial weakness (MRC 4 to 4+)  -van der Sluijs et al. 2003: Weakness of facial muscles other than external ocular musculature in 13/16 (81.3%)  -van der Sluijs et al. 2016b: 24/40 (60.0%) participants, no MRC severity reported  -Witting et al. 2014: **11/13 (84.6%)** participants with facial weakness |
|  |  | Sum of score ranging from 0 – 130 (n = 2) | Significant decline of total MRC score for 14 muscle groups over a period of 20 months, with two muscle groups regarding axial muscles (neck flexors and neck extensors) (Kroon et al. 2021, Kroon et al. 2022). |
|  |  | Number and % with weakness present (n = 1) | Axial muscle weakness (neck flexors > neck extensors) reported and ranging from mild (MRC 4) to moderate (MRC3) (Youssof et al. 2015). **Neck extensors: 3/27 (11.1%)** **Neck flexors: 9/27 (33.3%)** |
| **Quantitative Muscle Testing (QMT)** (n = 1) | **PerfO**: Portable dynamometer Citec CT 301 (n = 1) | Maximal isometric muscle contraction. Mean of three trials | **No weakness reported for neck flexors and neck extensors** **in the graph (data available in supplementary table) when compared to age- and sex-matched patients.** Some patients with a higher strength than reference values (max. 145% approximately) (Witting et al. 2014). No raw data presented in the study. No predilection of muscle impairment pattern. Weakness characterized by individual variations. |
| **Other test** (n = 1) | **PerfO**: Homemade standardized test (n = 1) | Capacity to raise head from bed for 5-6 seconds. | Incapacity to realize the test in **1/22 (4.6%)** patients (Tremblay-Tymczuk et al. 1992). |

**S2 Table 6**: Key findings of COA’s reporting **balance** **impairments**.

| **7 studies** | **COA** | **Method** | **Findings** |
| --- | --- | --- | --- |
| **PROM**: 1 study | | | |
| **Interview** (n = 1) | **PROM:** Semi-structured interview (n = 1) | « Framework » technic | Poor balance reported in **7/25 (28.0%)** patients (Kurtz et al. 2019). |
| **ClinRO:** 2 different studies | | | |
| **Clinical observation** (n = 2) | **ClinRO**: Physical examination (n = 2) | Standing capacity (n = 2) | Incapacity to stand independently in an 81 year-old man and a 33 year-old woman (Schmitt et al. 1981, Schober et al. 2001). |
|  |  | Stance on toes/heels + single-leg jump (n = 1) | Incapacity to raise on toes/heel and to jump on one leg in a 33 year-old woman (Schober et al. 2001). |
| **PerfO:** 4 different studies | | | |
| **Other tests** (n = 4) | **PerfO**: Motor Function Measurement (MFM) | **Part D1: standing and transfer tasks.**  Part D2: axial and proximal limb motor function Part D3: distal limb motor function  Lower score = more impairments (n = 4) | **Lower score reported for MFM part D1 in comparison to D2 and D3** (Fischmann et al. 2011, Kroon et al. 2021, Kroon et al. 2022)  **Significant decline** of MFM part D1 over a period of 20 months (Kroon et al. 2021, Kroon et al. 2022).  **No significant difference between patients and control for MFM total score (van der Sluijs et al. 2017).**  No precision regarding specific tasks altered from MFM part D1. |

**S2 Table 7**: Key findings of COA’s regarding walking short distances (d4500) limitations.

| **34 studies** | **COA** | **Method** | **Findings** |
| --- | --- | --- | --- |
| **PROM:** 6 different studies | | | |
| **Interview**  (n = 1) | **PROM:** Semi-structured interview (n = 1) | « Framework » technic | Walking capacity limitations (comfortable and maximal walking speeds) requiring the use of a walking aid in 6/25 (24.0%) of patients (Kurtz et al. 2019). |
| **Questionnaire**  (n = 5) | **PROM:** Neuro-QoL (n = 1) | Self-reported questionnaire in 17 domains, including physical, mental and social health. T-Score and comparison with normative data from a reference population. | Questionnaire selected to assess lower extremity function. “Mean Neuro-QOL score was 41.6 ± 9.6 (range 25.1-58.6), which is statistically significantly  lower than the mean of 50 ± 10 in the general US population (P<0.0001).”  Higher duration of lower limbs symptoms and use of walking aid were significantly correlated to a lower Neuro-QoL score. Neuro-QoL's score is correlated to SF-36 questionnaire’s score in a stronger fashion than the other variables. Neuro-QoL explains a large proportion of SF-36's physical domain (Youssof 2016). |
|  | **PROM:** Modified Rankin Scale (n = 1) | 0 to 5 scale. Higher score means patients more severely affected by OPMD disease and more dependant for mobility | 4/16 (25.0%) patients: score of 3/5 = moderate disability, requiring some help but no assistance required for walking. 3/16 (18.8%) patients: score of 4/5 = moderately severe disability, unable to walk without assistance and unable to attend their own body needs without assistance (Van Der Sluijs et al. 2003). |
|  | **PROM**: Home-made questionnaire about lower extremities (n = 1) | Use of walking? Which type of walking aid? | Use of walking aid or incapacity to walk independently was reported in **2/22 (9.2%)** patients. These patients used walking aid intermittently, primarily a cane (Tremblay-Tymczuk et al. 1992). |
|  | **PROM:** Sickness Impact Profile (SIP) (n = 2) | Measures functional impairments in different domains of functioning | SIP-136: Functional impairments were reported in **24/35 (69.0%)** patients in the « ambulation » domain of the SIP-136. The “ambulation” domain was the most frequently impaired domain in patients (van der Sluijs et al. 2016a).  SIP-68: Total SIP-68 score significantly higher in OPMD patients 3.6 (+/- 4.8) when compared to control group (0,1 (+/- 0,3). Significant higher score in OPMD patients in “physical mobility control” subscale (p = 0.006) (van der Sluijs et al. 2017). |
| **ClinRO:** 27 different studies | | | |
| **Clinical observation**  (n = 20) | **ClinRO:** Subjective analysis of walking pattern (n = 20) | Observation from a clinician without predetermined spatiotemporal parameters | *Walking capacity limitations (altered gait, use of walking aid during walking, assistance required and/or leg difficulties)* reported in **1/16 (6.3%)** to **6/14 (42.9%)** patients. Heterogeneity in gait parameters assessed and reported. No precision on methodological assessment of walking gait.  **Author’s last name, year: prevalence (%):**  -Alonsò-Pérèz et al. 2022: able to walk without a walking aid in 98 patients (90.7%) -Becher et al. 2001: abnormal gait in 17/49 (34.7%) -Blumen et al. 1993: Gait problem: 8/79 (10.1%) -Blumen et al. 2013: case study, difficulties walking in 1/5 (20.0%) -Bosch et al. 1979: case study, waddling gait -Fukuhara et al. 1982: case study, Waddling gait: 2/2 (100.0%) -Kuo et al. 2008: case study, Gait disturbance: 1/10 (10.0%) -Müller et al. 2001: case study, Waddling motion of the pelvis or unable to walk without assistance: 2/16 (12.5%) -Nishii et al. 2021: case study, 0/1 (0%) -Porschke et al. 1997: Impairment of walking often occurred, which rarely leads to the use of a wheel-chair (no prevalence reported) -Robinson et al. 2011: case study, 0/1 (0%) unsteadiness with a tendency to trip  -Ruëgg et al. 2005: case study, walk slowly due to fatigue; 1/1 (100.0%) -Saini et al. 2018: 0/4 (0%). All considered “independent” -Salvesen et al. 1996: 6/13 (46.2%) with walking limitations -Schmitt et al. 1981: 1/1 (100.0%) could not walk without any help -Schober et al. 2001: 2/2 (100.0%) waddling/rolling gait -Shan et al. 2014: For patients with limb weakness: All these cases were ambulatory at the last visit. -Tremblay-Tymczuk 1992: 2/22 (9.2%) One could walk with walking aid and one could not walk at all **-Van der Sluijs et al. 2017:** **Walking difficulties or other pelvic girdle or proximal leg motor symptoms 6/14 (42.9%)** -Vita et al. 1983: 6/12 (50.0%) when including family members  No walking limitation (Nishii et al. 2021, Robinson et al. 2011, Saini et al. 2018, Shan et al. 2014). |
| **Use of walking aid** (n = 11) | **ClinRO**: Recording of the use of a walking aid (n = 11) | Use of walking aid (yes/no, %) (n = 11) | Use of walking aid (cane > walker, roller, wheelchair) reported in **14/119 (12.0%)** to **40/89 (44.9%)** (Alonso-Jimenez et al. 2019, Alonso-Pérez et al. 2022, Brisson et al. 2020, Kurtz et al. 2019, Melkus et al. 2023, Salvesen et al. 1996, Tremblay-Tymczuk et al. 1992, Wallace et al. 2021, Witting et al. 2014, Youssof 2016, Youssof et al. 2015). *Brisson et al. 2020* Before 75 yo: 14.5% used a wheelchair (no distinction between outside or inside), 31.9% used any kind of walking aid. |
| **Patient file review** (n = 5) | Patient file review (n = 2) | Presence of walking limitation (yes/no, %) (n = 1) | *Alonsò-Pérèz et al. 2022* Canary cohort: - Normal walking: 98/108 (90.7%) - Use of walking aid: 8/108 (7.4%) - Total incapacity (wheelchair): 2/108 (1.9%)  European cohort:  - Normal walking: 77/119 (64.7%) - Use of walking aid: 32/119 (26.9%)  - Total incapacity (wheelchair): 10/119 (8.4%) |
|  |  | Presence of incapacity to walk a predetermined distance (n = 1) | *Brisson et al. 2020*  Incapacity to: - Walk a long distance (> 150 m): 126/315 (40.0%) - Walk a moderate distance (20 – 150 m): 91/314 (29.0%) **- Walk a short distance (< 20 m):** **55/322 (17.1%)**  Before 75 yo: 27.2% of patients were incapable to walk > 150 m without assistance. |
|  | Patient file review (n = 5) | Use of walking aid reported in medical file (n = 5) | Use of walking aid (cane, walker, roller, wheelchair) reported in 10/108 (9.3%) to 141/324 (43.5%) of patients (Alonso-Pérez et al. 2022, Becher et al. 2001, Brisson et al. 2020, Youssof et al. 2015).  Hip flexors weakness (MRC ≤ 3) associated with the use of a walking aid and lower mobility outcomes (with control of age of disease onset and comorbidities) (Youssof et al. 2015).  Brisson et al. 2020: Retrospective study, n=333 patients files. Before 75 yo: 31.9% used a walking aid. Women used a specialized equipment significantly later than men (Brisson et al. 2020).  MD Starnet website: 14/117 (12.0%) of OPMD patients were non-ambulant (Wallace et al. 2021). |
| **PerfO:** 5 different studies | | | |
| **Walk test**  (n = 2) | **PerfO**: 6-minute walk test (6MWT) (n = 2) | Maximal capacity test assessing the walking distance reached in six minutes in a 30 meters corridor | Significant difference between three age groups for reached distance (< 55 yo, 55 – 65 yo, > 65 yo). Tendency of mean distance reduction across age groups (Brisson et al. 2023).  **6MWT mean distance reached in meters** (n = 33): mean (SD) Total (n=33): 424 (117)  < 56 yo (n=11): 497 (78) 56 – 65 yo (n=10): 441 (80) > 65 yo (n=13): 343 (129)  *% from reference values:* No significant difference between 3 age groups. Total (n=33): **67.3 (17.0)**  < 56 yo: 71.4 (11.0)  56 – 65 yo: 71.5 (13.4) > 65 yo: 60.1 (22.3)  **No significant difference** in the distance reached during 6MWT between OPMD patients and controls (van der Sluijs et al. 2017).  Mean age of patients different between the two studies. |
|  | **PerfO:** 10-meter walk test (10mWT) (n = 1) | Confortable and maximal speed | *Comfortable speed (m/s) (n = 31)*: **no significant difference between three age groups** for comfortable speed (Brisson et al. 2023): Total (n = 31): 1.19 (0.31)  < 56 yo (n = 11): 1.29 (0.26)  56 – 65 yo (n = 10): 1.26 (0.30)  > 65 yo (n = 13): 1.06 (0.33)  *Maximum speed (m/s) (n = 33):* **significant difference between three age groups**. Walking speed tends to reduce with older age in OPMD (no statistical test) (Brisson et al. 2023): Total (n = 33): 1.57 (0.46)  < 56 yo (n = 11): 1.82 (0.45)  56 – 65 yo (n = 10): 1.64 (0.32)  > 65 yo (n = 13): 1.31 (0.47)  *10mWT comfortable* % from reference values: no significant differences between 3 age groups (Brisson et al. 2023) Total (n=31): **90.4 (21.9)**  < 55 yo: 94.1 (18.0)  55 – 65 yo: 95.0 (20.1) > 65 yo: 83.9 (25.9)  **10mWT maximal**  **No comparison to reference values for 10mWT maximal walking speed.** |
| **Other test** (n = 4) | **PerfO**: Timed Up & Go (TUG) (n = 1) | Time required to raise from a chair, walk 3 meters, turn around and sit back in the chair | Time to complete the TUG was **significantly higher in OPMD patients compared to controls, but values were still within normative values** (van der Sluijs et al. 2017)**.** Young sample of patients (mean age: 51.8 yo (39 to 63)) and MRC scale in lower limbs ranging from 4.5 to 5/5 (slight weakness only). |
|  | **PerfO**: Actometer (n = 1) | Mean number of walking acceleration per 5 min interval in 24 hours over 12 days | The number of accelerations per 5 min over a period of 24 hours **is similar between OPMD patients and controls**. Young sample of patients (mean age: 51.8 years old (39 - 63)) and MRC scale in lower limbs ranging from 4.5 to 5/5 (slight weakness only) (van der Sluijs et al. 2017). |
|  | **PerfO**: Motor Function Measurement (MFM) (n = 4) | Part D1: standing and transfer tasks.  **Part D2: axial and proximal limb motor function**  Part D3: distal limb motor function  Lower score = more impairments | No significant difference in OPMD patients for MFM total score and D2 and D3 subscales. Significant difference for D1 subscale between OPMD and controls (Fischmann et al. 2011).  **No significant decline over a period of 20 months for part D2 of the MFM (Kroon et al. 2021, Kroon et al. 2022).**  No significant difference between OPMD patients and controls for total MFM score (van der Sluijs et al. 2017).  Small to moderate sample sizes. Cross-sectional and prospective studies. |

**S2 Table 8**: Key findings of COA’s regarding changing basic body positions (d410) limitations.

| **16 studies** | **COA** | **Method** | **Findings** |
| --- | --- | --- | --- |
| **PROM:** 3 different studies | | | |
| **Interview**  (n = 1) | **PROM**: Semi-structured interview | « Framework » technic | Difficulties in rising from a chair reported in **2/25 (8.0%)** of patients. Difficulties in squatting or kneeling reported in 5/25 (25.0%) patients (Kurtz et al. 2019). |
| **Questionnaire**  (n = 2) | **PROM**: Home-made questionnaire | Capacity to raise from a chair independently, with or without supports, with the assistance of another person or total incapacity | Difficulties in rising from a chair or getting out of bathtub independently and requiring leaning or use of aid in **12/22 (54.6%)** of patients (Tremblay-Tymczuk et al. 1992, Van Der Sluijs et al. 2003). No precision regarding how the task is affected (handrests, leaning, support from a person or a walking aid). |
| **ClinRO:** 10 different studies | | | |
| **Clinical observation**  (n = 9) | **ClinRO**: Physical examination of transfer capacity (n = 7) | Capacity to raise from a chair independently, with support, with the assistance of another person or incapacity (n = 7) | Incapacity to raise from a chair without assistance reported in 3/16 (18.8%) to **13/22 (59.1%)** patients**.**  No precision regarding how the task is affected (handrests, leaning, support from a person or a walking aid, ...).  **Author’s last name, year: prevalence (%):**  -Bosch et al. 1979: case study, 1/1 (100.0%) difficulties raising from a chair  **-**Muller et al. 2001: case study, 3/16 (18.8%) raise from a chair -Salvesen et al. 1996: case study, 1/13 (7.7%) -Schmitt et al. 1981: case study, 1/1 (100.0%) **-Tremblay-Tymczuk 1992: 13/22 (59.1%)** 12 needed help and one unable -van der Sluijs et al. 2003: 9/16 (56.3%) -Vita et al. 1983: case study, 1/1 (100.0%) |
|  | **ClinRO:** Gower’s sign test (n = 4) | Observation of the capacity to stand from the ground. Use of hands to walk progressively through shins, knees and thighs = “+” | Positive Gower’s sign (use of hands to walk progressively through shins, knees and thigh) reported in **8/79 (10.1%)** to 3/16 (18.8%) of patients.  **Author’s last name, year: prevalence (%):**  **-Blumen et al. 1993: 8/79 (10.1%) Gower’s sign positive** -Fukuhara et al. 1982: case study, 1/1 (100.0%). Gower’s sign positive -Müller et al. 2001: case study, 3/16 (18.8%) Gower’s sign positive -Vita et al. 1983: case study, 1/1 (100.0%). Gower’s sign positive |
| **Patient file review** (n = 1) | Patient file review (n = 1) | Capacity to raise from a chair and from a crouched position (Gower’s sign) | Incapacity to raise from a chair reported in **35/320 (10.9%)** patients. Incapacity to stand from a crouch position (Gower’s sign test considered positive) reported in **97/294 (33.0%)** patients (Brisson et al. 2020). |
| **PerfO:** 5 different studies | | | |
| **Transfer test** (n = 1) | **PerfO:** 30 seconds Chair Stand Test (30s. CST) (n = 1) | Number of sit-to-stands realised in 30 seconds. One repetition = 1 complete sit-to-stand. | *Number of repetitions for 30s. CST (n = 34):* **significant difference between three age groups** (Brisson et al. 2023): Total (n = 34): 10 (7) reps < 55 yo (n = 11): 15 (6) reps 55 – 65 yo (n = 10): 11 (4) reps > 65 yo (n = 13): 6 (6) reps  *% from reference values (n = 34):* no significant difference between three age groups. Total (n = 34): **50.3 (30.1)**  < 55 yo: 65.7 (24.9)  55 – 65 yo: 55.3 (18.9)  > 65 yo: 33.4 (34.2) |
| **Other tests** (n = 4) | **PerfO**: Motor Function Measurement (MFM) (n = 4) | **Part D1: standing and transfer tasks.**  Part D2: axial and proximal limb motor function  Part D3: distal limb motor function  Lower score = more impairments | **Significantly lower MFM part D1 score** **in comparison to part D2 and D3** (Fischmann et al. 2011, Kroon et al. 2021, Kroon et al. 2022).  **Significant decline of MFM part D1 over a period of 20 months** (Kroon et al. 2021, Kroon et al. 2022).  **No significant difference** between OPMD patients and controls for total MFM score (van der Sluijs et al. 2017). |

**S2 Table 9**: Key findings of COA’s regarding going up and down stairs (d451) limitations.

| **17 studies** | **COA** | **Method** | **Findings** |
| --- | --- | --- | --- |
| **PROM**: 3 different studies | | | |
| **Interview**  (n = 1) | **PROM:** Semi-structured interview | « Framework » technic | Difficulties in climbing steps, stairs or curbs in 17/25 (68.0%) of patients (Kurtz et al. 2019) |
| **Questionnaire**  (n = 2) | **PROM**: Home-made questionnaire (n = 2) | Capacity to use stairs independently, with supports, with assistance from another person or total incapacity | Difficulties in using stairs (ascend > descend) with or without the use of handrails or a cane reported in **8/22 (36.4%)** to **13/16 (81.3%).** Total incapacity reported in one patient (4.6%) (Tremblay-Tymczuk et al. 1992, Van Der Sluijs et al. 2003). |
| **ClinRO:** 10 different studies | | | |
| **Clinical observation** (n = 9) | **ClinRO**: Clinical observation of use of stairs capacity (n = 9) | Capacity to climb stairs | Difficuties climbing stairs reported in up to 3/16 patients (18.8%) when including case studies**.** No descent of stairs assessment. No precision regarding how the incapacity is present (use of handrails, gait pattern, pauses required). No precision regarding climbing speed.  **Author’s last name, year: prevalence (%):**  -Blumen et al. 2013: case study, 1/5 (20.0%) difficulty climbing stairs -Bosch et al. 1979: case study, 1/1 (100.0%) difficulty climbing stairs -Fukuhara et al. 1982: case study, 1/2 (50.0%) weakness at hips that interfered with going up and down stairs -Kuo et al. 2009: case study, 1/10 (10.0%) gait disturbance including climbing stairs **-Muller et al. 2001:** case study, **3/16 (18.8%)** difficulties climbing stairs -Nishii et al. 2021: case study, 1/1 (100.0%) required assistance to climb stairs -Porschke et al. 1997: no prevalence reported among 43 patients, but limb girdles impairing the ability to climb stairs reported among the consequences -Salvesen et al. 1996: case study, 2/13 (15.4%) difficulties climbing stairs -Vita et al. 1983: case study, 1/12 (8.3%) difficulties climbing stairs |
| **Patient file review** (n = 1) | Patient file review (n = 1) | Review of the capacity to use stairs in patients' medical file | Incapacity to climb stairs independently reported in **76/302 (25.1%)** patients and **incapacity reported before 75 yo in 14.5% over the 25.1% of participants (more than half of the participants presenting the incapacity)** (Brisson et al. 2020). |
| **PerfO:** 4 different studies | | | |
| **Stair test**  (n = 4) | **PerfO**: 10 steps stair test (n = 4) | Time required to ascend or descend 10 steps | 10-step stair test: total incapacity to climb stairs reported in **3/16 (18.8%)** to **9/43 (20.9%)** of patients (Kroon et al. 2021, Kroon et al. 2022, Witting et al. 2014).  **Significant decline of 10 steps stair test (climb) performance over a period of 20 months** (Kroon et al. 2021, Kroon et al. 2022).  In a cross-sectional study: 1/16 (6.3%) patient had a slower time to realize the 10 steps stair test (12 sec. vs 8 sec.) (Witting et al. 2014).  *10 steps stair test comfortable climb, maximal climb, comfortable descent and maximal descent (seconds):* **significant difference between three age groups** (< 55 yo, 55 – 65 yo and > 65 yo) for the time required to execute the test (Brisson et al. 2023):  *% from reference values 10 steps max climb (n = 32)* Total (n = 32): **65.5 (28.0)** < 56 yo: 85.1 (24.4)  56 – 65 yo: 66.3 (18.1)  > 65 yo: 45.1 (25.8)  *% from reference values 10 steps max descent (n = 32)* Total (n = 32): **70.0 (23.8)** < 56 yo: 84.4 (22.3)  56 – 65 yo: 71.1 (16.0) > 65 yo: 54.7 (23.1) |
