# Supplementary material for: What is known about muscle weakness, balance impairments and indoor mobility limitations in oculopharyngeal muscular dystrophy? A scoping review
Source: J Neuromuscul Dis. 2025 Dec 30:22143602251397052. Online ahead of print. doi: 10.1177/22143602251397052 (PMC13434899; doi:10.1177/22143602251397052)
Supplement: Supplementary file 3 [file sj-docx-4-jnd-10.1177_22143602251397052.docx]

**Appendices**

**Appendix A**: Complete search strategy

("muscular dystrophy, oculopharyngeal"[MeSH Terms] OR ("muscular"[All Fields] AND "dystrophy"[All Fields] AND "oculopharyngeal"[All Fields]) OR "oculopharyngeal muscular dystrophy"[All Fields] OR ("oculopharyngeal"[All Fields] AND "muscular"[All Fields] AND "dystrophy"[All Fields]) OR ("Muscular Dystrophies"[MeSH Terms] AND "oculopharyngeal"[All Fields])) AND ((("motor"[All Fields] OR "motor s"[All Fields] OR "motoric"[All Fields] OR "motorically"[All Fields] OR "motorics"[All Fields] OR "motoring"[All Fields] OR "motorisation"[All Fields] OR "motorised"[All Fields] OR "motorization"[All Fields] OR "motorized"[All Fields] OR "motors"[All Fields]) AND "impair*"[All Fields]) OR ("muscle s"[All Fields] OR "muscles"[MeSH Terms] OR "muscles"[All Fields] OR "muscle"[All Fields]) OR "mobilit*"[All Fields] OR ("balance"[All Fields] OR "balanced"[All Fields] OR "balances"[All Fields] OR "balancing"[All Fields]) OR (("functional"[All Fields] OR "functional s"[All Fields] OR "functionalities"[All Fields] OR "functionality"[All Fields] OR "functionalization"[All Fields] OR "functionalizations"[All Fields] OR "functionalize"[All Fields] OR "functionalized"[All Fields] OR "functionalizes"[All Fields] OR "functionalizing"[All Fields] OR "functionally"[All Fields] OR "functionals"[All Fields] OR "functioned"[All Fields] OR "functioning"[All Fields] OR "functionings"[All Fields] OR "functions"[All Fields] OR "physiology"[MeSH Subheading] OR "physiology"[All Fields] OR "function"[All Fields] OR "physiology"[MeSH Terms]) AND "capacit*"[All Fields])) AND ("human s"[All Fields] OR "humans"[MeSH Terms] OR "humans"[All Fields] OR "human"[All Fields])

**Appendix B:** PRISMA-ScR organizational flow chart

 

**Appendix C**: Final version of the extraction grid

| **Article details** | Article 1 | Article 2 … |
| --- | --- | --- |
| Title |  |  |
| Country |  |  |
| Design |  |  |
| Objectives |  |  |
| Rationale |  |  |
| Measurement tools |  |  |
| Study eligibility and participants inclusion |  |  |
| **Population** | Article 1 | Article 2 … |
| Number of participants |  |  |
| Age (mean), years |  |  |
| Age (min., max.), years |  |  |
| Symptoms or disease duration (mean and min-max), years |  |  |
| Age and symptoms at examination (mean or median, number and %) |  |  |
| Age at diagnosis (mean or median, number, min-max and range), years.  PR = patient reported. MR = in Medical Record CR = clinicaly reported |  |  |
| Age of first symptom onset (mean or median (Md), min-max and range), years.  PR = patient reported. MR = in Medical Record CR = clinically reported |  |  |
| Ethnicity |  |  |
| Sex (n, %) |  |  |
| Comorbidities (type, n, %) |  |  |
| Inclusion and exclusion of participants |  |  |
| Number of (GCN) expansion |  |  |
| Other |  |  |
| **Motor impairments** | Article 1 | Article 2 … |
| Axial muscle weakness (neck, facial, oculomotor, trunk and abdominal muscles) |  |  |
| Proximal lower limb muscle weakness (hip and knee) |  |  |
| Proximal upper limb muscle weakness (shoulder, scapula and elbow) |  |  |
| Upper and/or lower distal limb muscle weakness (wrist, ankle) |  |  |
| **Mobility limitations** | Article 1 | Article 2 … |
| Short distance walking (d4500) |  |  |
| Changing basic body positions (d410) |  |  |
| Going up and down stairs (d451) |  |  |
| Other |  |  |
| Comments |  |  |
